# Subcutaneous and orally self-administered high-dose carprofen shows favorable pharmacokinetic and tolerability profiles in male and female C57BL/6J mice

**DOI:** 10.3389/fvets.2024.1430726

**Published:** 2024-09-20

**Authors:** Aylina Glasenapp, Jens P. Bankstahl, Heike Bähre, Silke Glage, Marion Bankstahl

**Affiliations:** ^1^Institute for Laboratory Animal Science and Central Animal Facility, Hannover Medical School, Hannover, Germany; ^2^Department of Nuclear Medicine, Hannover Medical School, Hannover, Germany; ^3^Department of Pharmacology, Research Core Unit Metabolomics, Hannover Medical School, Hannover, Germany; ^4^Department of Biological Sciences and Pathobiology, Pharmacology and Toxicology, University of Veterinary Medicine Vienna, Vienna, Austria

**Keywords:** pharmacokinetics, analgesia, carprofen, mice, tolerability, behavioral pain indicators, subcutaneous, drinking water

## Abstract

**Introduction:**

Surgical interventions in mice require appropriate pain relief to ensure animal welfare and to avoid influence of pain on research findings. Carprofen is a non-steroidal anti-inflammatory drug commonly used as an analgesic for interventions inducing mild to moderate pain in laboratory rodents. Despite its frequent use, species-specific data on pharmacokinetics (PK), side effects, and potential impact on behavioral pain indicators are limited.

**Methods:**

We determined PK and tolerability profiles of carprofen in healthy male and female C57BL/6J mice (*n* = 42), administered at highest recommended doses via single subcutaneous (s.c.) injection (20 mg/kg) and oral self-administration (25 mg/kg/24 h) per drinking water (d.w.) for 5 days. Plasma concentrations were measured at various time points after the start of the treatment (*n* = 6 per time point), and side effects were evaluated using a modified Irwin test battery, hematology, and histopathology. Additionally, potential interference with cage-side behaviors commonly used for pain assessment, such as the mouse grimace scale, wheel running, burrowing, nesting, and grooming activity, was investigated.

**Results:**

Maximum plasma concentrations of 133.4 ± 11.3 μg/ml were reached 1 h after single s.c. injection with an elimination half-life of 8.52 h. Intake from d.w. resulted in a steady state within 24 h after the start of the treatment with plasma levels of around 60 μg/ml over 5 days in both sexes. The medicated water was well-accepted, and increased d.w. intake was observed in the first 24 h after exposure (*p* < 0.0001). The Irwin test revealed only minor influence on tested behavior and physiological functions. However, during treatment via d.w., an increase in body temperature (*p* < 0.0001) was observed, as well as a reduction in voluntary wheel running activity by 49–70% in male mice. Moreover, grooming behavior was slightly affected. Hematology and histopathology were without pathological findings that could be attributed to carprofen treatment. High-dose carprofen can be considered safe and of favorable PK for both administration routes assessed in healthy C57BL/6J mice of both sexes. Further efficacy evaluation of carprofen as monoanalgesic or component of multimodal post-surgical regimens is clearly encouraged; however, the impact on behavioral markers used for pain assessment should be considered in this context.

## 1 Introduction

Numerous experimental animal studies include surgical interventions, entailing the need for effective post-operative pain relief. Nevertheless, structured literature reviews suggest that peri- and post-operative pain in laboratory rodents is often undertreated (1–3). However, refinement and tailoring of pain management are hampered by largely lacking pharmacokinetic (PK) and tolerability profiles in laboratory rodents, even for standard analgesic drugs.

For pain therapy after surgical interventions in mice, an effective and well-tolerable analgesic would be desirable, with stress-free administration for animals and convenient to apply in daily routine. Here, carprofen might be a promising candidate for subcutaneous (s.c.) injection but also for oral self-administration via drinking water (d.w.), as it is stable in water for at least 7 days (4, 5) and there is no indication for potential taste-based aversion to carprofen-medicated water (4, 6, 7).

Carprofen is a non-steroidal anti-inflammatory drug and widely used both for the management of chronic pain as well as acute soft tissue injury in animals (8, 9). It is licensed for veterinary use as racemic mixtures of R- (–) and S- (+) enantiomers; however, so far no formulation has been approved for mice. It exerts its analgesic effects through inhibition of cyclooxygenase (COX) isomers, but species differences for its PK, COX-2 selectivity, and enantiomer potency have been reported (10). In laboratory mice, carprofen is often used to attenuate mild to moderate pain, but there is only limited knowledge about plasma concentrations obtained in this species, especially for administration via drinking water, and so far only at doses that are considered to provide limited analgesic efficacy (4, 6, 11, 12). While new PK information has recently become available on female CD1 mice for s.c. injection (13), PK data on mice of the most widely used inbred strain, C57BL/6, are still rather little. Data about tolerability are even more sparse. Currently, only a few reports on side effects are available for mice (10), but recent reports indicate an increased risk of gastrointestinal ulceration in female CD1 mice (14). As pain therapy should reduce rather than increase post-operative distress, a structured assessment of potential adverse effects on clinical, blood, and histological levels is essential in order to improve pain therapy protocols.

Spontaneous home-cage behavior of mice, such as voluntary wheel running, burrowing, nesting, and grooming activity, or mouse grimace scale (15), is increasingly assessed after surgical interventions as surrogate measures of pain and analgesic efficacy. However, potential confounding effects of carprofen treatment on cage-side behaviors have not been investigated so far, although respective knowledge is of importance in order to differentiate pain-induced behavioral changes from those mediated by the analgesic drug itself.

Therefore, the first major aim of this study was to provide species- and strain-specific PK and tolerability profiles for high-dose carprofen after s.c. and prolonged self-administration via d.w. in C57BL/6J mice to serve as solid orientation for refinement of carprofen-based pain management protocols. The second objective was to reveal potential impact of carprofen treatment on established behavioral surrogate markers of post-operative pain.

## 2 Material and methods

### 2.1 Animals, health monitoring, and cage setup

This study was approved by the responsible state authority (Niedersächsisches Landesamt für Verbraucherschutz und Lebensmittelsicherheit) under reference number 33.8-42502-04-21/3640. C57BL/6J mice were bred in-house in an IVC system. After transfer to the experimental room, mice were housed in experimental groups of three female or male individuals per cage (type 3, macrolone, UNO BV, The Netherlands) in an open (conventional) cage system on standard bedding material (wood chips from spruce, poplar, and aspen trunks, LAB.BED, Thomsen Räucherspäne Räucherholz GmbH & Co. KG, Germany) in a temperature-controlled facility (average temperature 20.8°C, average humidity 48.0%) under a 14/10 h light/dark cycle (lights on: 6:30 a.m./lights off: 8:30 p.m.) with food (altromin 1320 standard diet, Altromin Spezialfutter, Germany) and filtered (particle filter, 5 μm pore size) tap water (non-acidified) *ad libitum*. As sex-based differences in pharmacokinetics, nociception, and behavior have been reported, both sexes were included in our study (15–17). Routine health monitoring according to FELASA recommendations (18) did not reveal any evidence of infection with common murine pathogens except for murine astrovirus, *Pneumocystis murina, Rodentibacter* sp., and apathogenic intestinal flagellates (see Supplementary material for further details). Experimental group size included 21 male and 21 female mice. As control for histologic analysis and blood profiles, six male and six female age-matched C57BL/6J mice kept under the same conditions were used. At the start of experiments with age of 12 weeks, mice weighed 28.0 ± 1.3 g (male) and 21.9 ± 1.2 g (female). Mice were transferred from the breeding area to the experimental room 4 weeks before the start of the experiments. This time was subdivided into 1 week of adaptation phase to the new room without particular handling and 3 weeks of habituation to all handling procedures. Each mouse was adapted three times to weighing, manual fixation, restrainer fixation, and tail immersion test (for tail immersion test habituation, lukewarm water was used). In their home cages, the mice had continuous access to nesting material, running disks installed on plastic houses, and wooden gnawing material as additional enrichment (plexx B.V. Aspen Bricks S, the Netherlands). A detailed cage and running wheel setup is provided in Supplementary Figure 7.

### 2.2 General experimental design and blood sampling procedure

**Figure 1:**
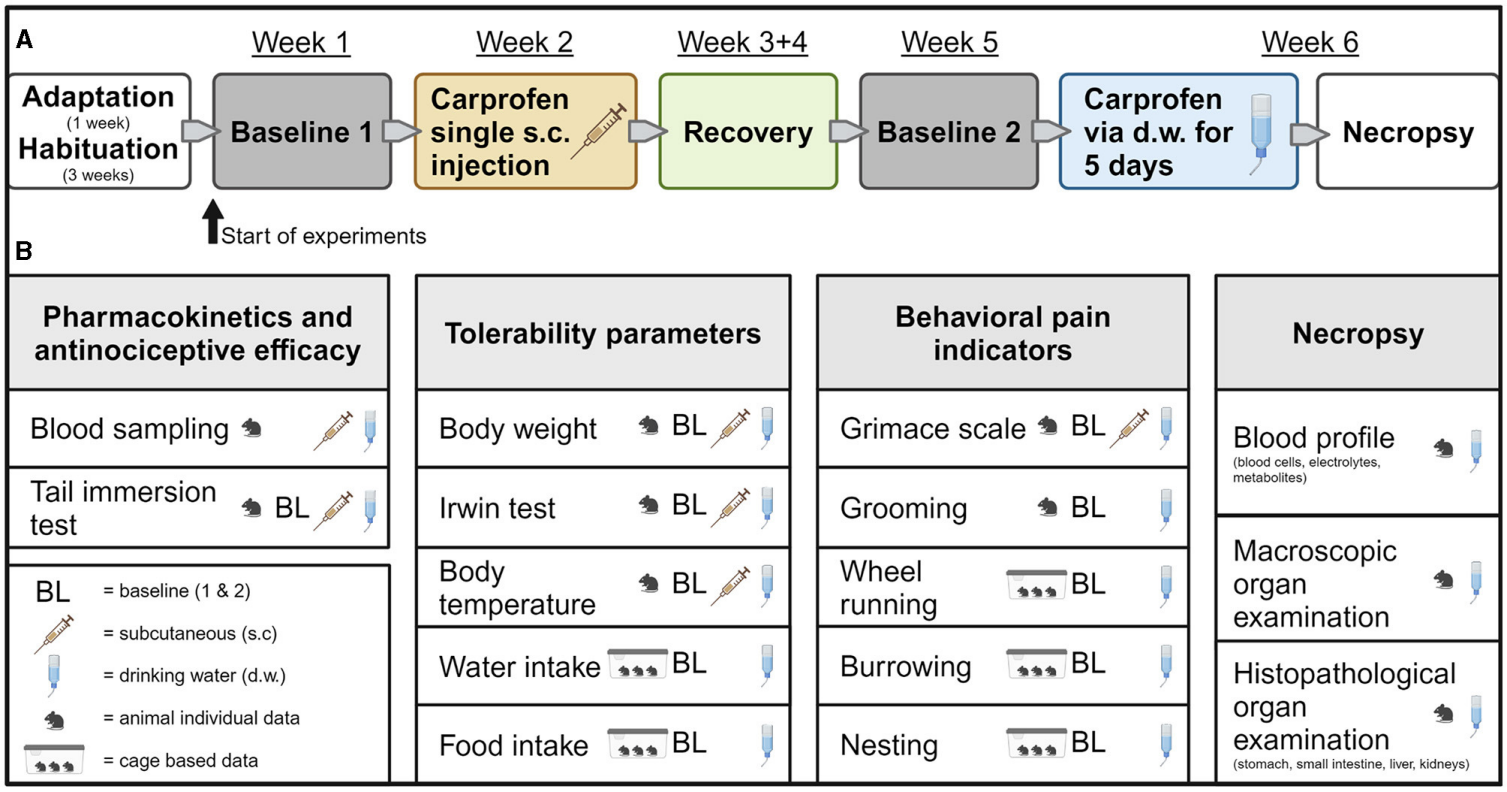
Experimental design. **(A)** Timeline of complete experimental setup. **(B)** Overview of all interventions and parameters. Created with BioRender.com.

An overview of the experimental setup is provided in Figure 1. Animals were habituated to all handling procedures and interventions and were trained for burrowing before experiments started. Running disks were accessible constantly from the beginning of habituation. During baseline phase 1 (BL1), data of all parameters of interest were obtained over 5 days, including food and water consumption, body weight, nest score, Irwin test, mouse grimace scale, body temperature, tail immersion test, wheel running, and burrowing activity. This was followed by s.c. injection and subsequent blood collection for PK analysis at 1, 2, 3, 6, 12, 24, and 48 h post-injection. After 2 weeks of recovery, a second baseline phase (BL2) was performed identical to BL1. In weeks 3 and 4 (recovery phase), each mouse was monitored and handled two times per week, including body weight measurement, clinical scoring, and renewal of identification by pen marking on the tails. Detailed habituation was not resumed during this phase. Cage equipment remained as before. Then, carprofen was administered via d.w. for 5 days, and blood was collected at 3, 6, 12, 24, 36, 108, and 120 h after the start of administration. Blood was sampled from a lateral tail vein by scalpel micro-incision, which has been shown to be of lower burden to the animals than withdrawal from the facial vein or the retro-orbital sinus (19). Blood was collected in EDTA tubes (Sarstedt 200 μl Microvette^®^, Germany) at all sampling time points. At each collection time point (see above), samples (~120 μl) were taken from six mice (three male, three female). Due to organizational reasons, the number of animals is deviant for s.c. treatment after 1 h (two male, one female), 2 h (four male, three female), and 3 h (three male, five female). No additional animals were added for 1 h, as an influence on outcome was considered irrelevant. Immediately after sampling, blood-containing tubes were placed on ice, then centrifuged (2,500 × *g*/10 min/4°C), and plasma was stored at −80°C until further processing. Directly prior to blood sampling, tests for tolerability (Irwin test) and nociceptive efficacy (tail immersion test) were conducted in the respective six mice. At the end of the experiment, mice were killed by CO_2_ inhalation and subsequent cardiac puncture (20). This euthanasia method allows for the mice to stay in their home cage without additional handling stress. The flow rate used was low and adjusted to the volume of the home cage (21). Final blood samples were used for full blood count and analysis of electrolytes, glucose, and lactate. Furthermore, necropsy of each animal, including macroscopic investigation for abnormalities, was performed. The stomach, small intestine, liver, and kidneys were dissected for histological processing.

### 2.3 Carprofen dosing and administration procedure

The carprofen dosage both for s.c. and oral administration was chosen based on the current GV-SOLAS expert information on pain management for laboratory animals (22). Of the suggested dose ranges for mice (5–20 mg/kg every 12 h s.c. or 10–25 mg/kg/d via the d.w.), the highest doses were applied. A commercially available carprofen solution (Zoetis, Rimadyl^®^ 50 mg/ml, injection solution for dogs and cats) was diluted with 0.9% sterile sodium chloride (B. Braun, Germany) to achieve a 10 ml/kg injection volume. The s.c. injection dose was 20 mg/kg. For d.w. administration, the injection solution was diluted in filtered tap water (non-acidified) targeting a carprofen dose of 25 mg/kg/24 h. Carprofen concentration calculation for group-housed mice (three mice/cage, seven cages per sex) was based on mean water consumption/24 h measured over 5 days (BL 2; male mice, 23.4 ml; female mice, 17.5 ml) and mean body weight (male mice, 29.9 g; female mice, 23.4 g). Carprofen-medicated d.w. was initially offered between 7:00 and 8:00 a.m. and provided continuously for five consecutive days. The medicated water was freshly prepared every morning in red polyphenylsulfon water bottles (Tecniplast, Italy). As carprofen-medicated water is reported to be readily consumed by mice (4), no habituation was performed.

### 2.4 Sample preparation and liquid chromatography-mass spectrometry analysis

Carprofen content in plasma was quantified by liquid chromatography-mass spectrometry (LC-MS/MS). Plasma samples and analgesic calibrators (25 μl) were thawed and diluted using 100 μl extraction solvent (acetonitrile/methanol 1/2) containing 12.5 nM efavirenz (obtained from the NIH AIDS Research and Reference Reagent Program, Division of AIDS, NIAID, NIH) as internal standard (equals a final concentration of 10 nM/sample) in a 1.5 ml reaction tube (SafeSeal^®^, Sarstedt, Germany) for analyte extraction and protein denaturation. Samples were mixed for 30 s using a vortex mixer and frozen overnight at −20°C to complete protein precipitation. Then, samples were thawed and centrifuged for 10 min at 20,800 × *g*/4°C for protein separation. For mass spectrometry analysis, samples were diluted 1:500 using dilution solvent (acetonitrile/methanol/water 2/2/1) containing 10 nM internal standard, and 100 μl were transferred into mass spectrometry vials (Wicom, Heppenheim, Germany) with inserts (Macherey-Nagel, Düren, Germany) for carprofen quantification. This involved chromatographic separation on a reversed-phase C18-column (ZORBAX Eclipse XDB-C18 1.8 μ, 50 × 4.6 mm, Agilent, Santa Clara, California, USA) connected to a C18 security guard (Phenomenex, Aschaffenburg, Germany), which was kept at 40°C during the whole analysis. A linear gradient was performed using an HPLC system consisting of two LC-30AD HPLC pumps, a SIL-30AC temperature-controlled autosampler, a DGU-20A5 degasser, a CTO-20AC oven, and a CBM-20A control unit (Shimadzu, Duisburg, Germany). Ten microliter of the sample was injected. Elution started with 80% of solvent A (water + 0.1% formic acid). Within 7 min, the amount of solvent B (methanol + 0.1% formic acid) was linearly increased to 95%. This composition was maintained for 3 min followed by a 3-min re-equilibration of the column back to 80/20 (solvent A/solvent B). The total analysis time was 13 min at a flow rate of 0.4 ml/min. The retention time of carprofen as well as efavirenz (internal standard) was 7.7 min. Analytes were detected by triple quad mass spectrometry (5500QTRAP; Sciex, Framingham, Massachusetts) in multiple reaction monitoring (MRM) mode. Ionization was achieved using negative electrospray ionization at 650°C. For carprofen, the mass transition m/z 272 → 226 was optimized for quantification, and for efavirenz, the mass transition m/z 314 → 244 was used. Control of HPLC and the mass spectrometer as well as data sampling was performed by Analyst software (version 1.7, Sciex). For quantification, calibration curves were created by plotting peak area ratios of carprofen, and the internal standard vs. the nominal concentration of seven calibrators containing 15.6–1,000 nM carprofen (corresponding to a lower limit of quantification of 0.01 μg/ml and an upper limit of quantification of 0.27 μg/ml) prepared in mouse plasma. The calibration curve was calculated using quadratic regression and 1/ × weighing.

Pharmacokinetic analysis was performed by using Phoenix WinNonlin [Version 8.2, Certara, Princeton, NJ, WinNonlin (RRID:SCR_024504)]. Non-compartmental analysis was applied for the determination of the plasma elimination half-life of carprofen.

### 2.5 Tolerability assessment and side effects

#### 2.5.1 Irwin test battery, body temperature, and mouse grimace scale

Prior to blood sampling, tolerability of carprofen was examined by a modified Irwin test battery (23) at 1, 2, and 3 h after s.c. injection and at 3, 6, 12, 24, 36, 108, and 120 h after the start of voluntary uptake via d.w. The Irwin test is a behavioral test battery widely used by the pharmaceutical industry to determine whether subjects show adverse effects from candidate compound treatment. The test included observations without handling, followed by assessments in an open field, followed by handling-based assessment of parameters (see Supplementary Table 1 for parameters assessed). For analysis, parameters were assigned to four different categories, i.e., excitation, coordination, sedation, and autonomous system. The mouse grimace scale was scored as part of the Irwin test assessment and visually assessed (without handling of the mice) in the home cage (24, 25) according to the established mouse grimace scale score (26). Immediately after the Irwin test procedure, rectal body temperature was measured (PhysioSuite^®^ for mice and rats, Kent Scientific Corporation, USA). For visualization of the Irwin test, a heat map was created showing the percentage of changes per investigated time point after treatment (*n* = 3 male and 3 female per time point, except s.c. treatment after 1 h (two male, one female), 2 h (four male, three female), and 3 h (three male, five female). For further analysis of the Irwin test outcome, a sum score of each individual mouse and test category (excitation, coordination, and sedation) was generated. Total score values were added for a total sum score. Negative score values were handled as positive score values for addition to sum score. Test parameters for which presence or absence were recorded were given 2 points if they deviated from normal. Handling-associated vocalization was compared to animal-individual BL and assessed with 2 points when deviant from BL. Vocalization during reflex testing (eyelid, pinna) was scored with 2 points when present. The presence of unusual behavior documented in free text (fasciculation) was scored with 2 points per observed behavior.

#### 2.5.2 Food and water intake and clinical score

Food and water consumption were gravimetrically measured per cage every 24 h during baseline phases (BL1 and BL2). During substance administration via d.w., water bottles were additionally weighed at blood sampling time points (for respective cages), resulting in fluid consumption data for day and night. Clinical score was determined two times a week during baseline phases (BL1 and BL2) and daily during carprofen administration phases. Clinical scoring included judgment of activity, general state of health, behavior, body posture, and body weight (Supplementary Figure 1A).

#### 2.5.3 Cage-side behaviors other than grimace scale

A variety of behavioral parameters, such as burrowing, nesting, and grooming behavior, and wheel running activity, have been suggested as potential indicators of pain in laboratory mice (15). They were included in this study to determine a potential impact of carprofen treatment on their outcome. To avoid impact of cage change on cage-side behaviors, cages were changed already 2–3 days prior to the start of assessment (BL1 and BL2, carprofen via d.w.). Nesting and gnawing materials were provided continuously throughout the whole experiment.

#### 2.5.4 Voluntary wheel running

Commercially available angled mouse running discs and igloos were purchased from ZOONLAB GmbH (Castrop-Rauxel, Germany). Running time (h:min:s), running distance (km), average velocity (km/h), and maximum velocity (km/h) were recorded as previously described (27–29) by bike computers (Sigma BC 16.16 STS, SIGMA-ELEKTRO GmbH, Germany). In brief, the sensor was fixed on the outside of the cage in close proximity (< 1 cm) to the magnet (neodymium, 10 × 3 mm), which was glued to the running disc. To avoid any accidental shifting of the igloos, the position of the setup in the cage was secured by a customized pedestal (polyethylene), which was attached to the cage floor with adhesive tape (Supplementary Figure 7). Wheel running parameters were continuously recorded and read out in the morning between 8:00 and 9:00 a.m. to obtain data during the “night” phase (lights on at 06:30 a.m.) and in the afternoon between 4:00 and 6:00 p.m. to record running behavior during “day” phase (lights off at 08:30 p.m.) over five consecutive days. Mice had continuous access to running discs during the complete experimental period. The first BL measurement in this study was performed 1 week after equipment of home cages with running disks. Data were recorded two times for baseline (BL1 and BL2) and under carprofen treatment. Only the second baseline (BL2) recording was used as reference for data analysis.

#### 2.5.5 Burrowing activity

Burrowing procedure was performed to investigate motivated goal-directed behavior (15) during substance administration according to a modified protocol from Deacon (30). For assessment of burrowing behavior, empty transparent bottles (volume 330 ml, length 15 cm, diameter 5 cm, polycarbonate) were filled with food pellets (altromin 1320 standard diet, Altromin Spezialfutter, Lage, Germany). First, mice were habituated two times to burrowing overnight by placing food pellet-filled bottles in the front left corner of the cage. Next morning, bottles and food were removed. For experiments, bottles were filled with 151 ± 2 g (mean ± SD) food pellets, placed in the cage, and removed after 2 h. Latency to start of burrowing (min) was visually measured for the first 30 min. In cases where the mice started burrowing later, 30 min was used as value for data analysis. At the end of burrowing procedure (after 2 h), the remaining pellets in the tube were weighed and the amount burrowed (%) was calculated. Burrowing procedure was performed between 11:00 a.m. and 1:00 p.m. according to a previously described protocol (31). Two baseline data sets (BL1 and BL2) were recorded for each cage; BL2 was used for statistical analysis. During oral substance administration, burrowing behavior was assessed every day for 2 h (at 24, 48, 72, and 96 h).

#### 2.5.6 Nest building

Nesting behavior was observed to detect alterations of intrinsically motivated behavior under drug treatment according to the established nesting score (6): 1 = no cotton pieces grouped together; 2 = cotton pieces paired together in one or two pairs; 3 = 3 cotton pieces grouped together; 4 = all cotton pieces grouped together; 5 = all four cotton pieces grouped together and completely shredded. Assessment of nest building test was performed using four cotton rolls (Ø 12 mm × 37 mm, ANT Tierhaltungsbedarf, Germany) per cage (Supplementary Figure 8A). Existing nesting material was taken out, and four new cotton rolls were provided 10 h after the start of carprofen treatment via d.w., and the nest score was recorded for the first time 2 h later. Subsequently, nests were scored every day in the morning (8:00–9:00 a.m.; 14, 38, 62, 86, 110 h after administration of nesting material) and in the afternoon (4:00–6:00 p.m.; 24, 48, 72, 96 h after administration of nesting material) (6). Two individual baselines (BL1 and BL2) of nesting were performed for statistical analysis, the median and range of BL2 are displayed.

#### 2.5.7 Grooming transfer test

Grooming behavior as potential indicator for pain or distress was evaluated according to Oliver et al. (6). Two baseline trials (BL1 and BL2) were performed. For statistical analysis, the median and range of BL2 are displayed. At 10 h after the start of carprofen administration via d.w., 8 μl of fluorescent GloGerm Oil (Glo Germ Company Utah, USA) was applied onto the skin of the neck region (first trial). When all mice had reached the highest possible score value (score 5), the fluorescent was applied again, and a second trial was performed. Using an UV flashlight (UVL 1006, Glo Germ Company Utah, USA), 2 h after application, and then every day in the morning between 8:00 and 9:00 a.m. (14, 38, 62 h after application), fluorescent oil distribution was scored according to an established grooming score (6) (Supplementary Figure 8B): 1 = fluorescence is strong at the application site on the forehead between the ears; 2 = fluorescence is present at the application site and front and/or rear nails; 3 = fluorescence is at the application site and the ears; signal may be present at front and/or rear nails; 4 = fluorescence is absent from the nails and ears but the traceable amount remains at application site; 5 = fluorescence is no longer visible. Two female mice with alopecia in the neck region were excluded from analysis.

### 2.6 Anti-nociceptive efficacy

Anti-nociceptive activity of NSAIDs has been detected before in the tail immersion test (32, 33), but not for carprofen. Two baseline measurements (BL1 and BL2) were performed for each individual mouse as a basis for evaluation of the anti-nociceptive efficacy of carprofen. After carprofen treatment, the test was always performed prior to the blood sampling procedure (three male and three female mice per time point). A water bath maintained a constant temperature of 50.1 ± 0.3°C (*n* = 167) for thermal stimulus. During the procedure, mice were placed into a customized cylindric restrainer (polyvinyl chloride) equipped with a cutout for the mouse tail. The distal 3 cm of the tail was marked and subsequently immersed in hot water and time until reflex reaction was manually stopped (stop watch ROTILABO^®^, Carl ROTH, Germany; seconds, two decimal places). This was repeated three times with < 20-s interval between measurements (34). To prevent potential heat-induced skin damage when showing no reflex reaction, the tail tip was removed from hot water after 10-s cut-off time. The mean of the three measures was built and used for statistical analysis. For statistical analysis, ratios of baseline measurements were compared to the ratio of baseline to individual treatment for each animal (BL2/BL1 vs. carprofen s.c./BL1; BL1/BL2 vs. carprofen d.w./BL2) using the Kruskal–Wallis test followed by multiple comparisons test. For graphic illustration, ratios between individual baseline and treatment for each animal were used (carprofen s.c./BL1; carprofen d.w./BL2).

### 2.7 Final blood analysis

Despite some concern about animal welfare using CO_2_ inhalation as euthanasia method, there is insufficient evidence for unbiased assessment of this euthanasia method on welfare indicators in laboratory mice (35). Still, euthanasia by CO_2_ inhalation is an accepted and commonly used method, which is in accordance with Annex IV of the directive 2010/63/EU and conforms to the most recent “AVMA guidelines for the euthanasia of animals: 2020 edition” (20). Here, after CO_2_-induced asphyxiation, performed in an AVMA-approved humane method in the animal's home cage (20), blood from the final cardiac puncture was sampled in EDTA tubes (Sarstedt 500 μl Microvette^®^, Germany) and immediately stored on ice until analysis to generate full blood count (scil Vet abc, scil animal care company GmbH, Germany), including leukogram of white blood cells (WBC), lymphocytes (LYM), monocytes (MO) and granulocytes (GRA). Red blood cell (RBC) count includes hemoglobin (HGB), hematocrit (HCT), platelets (PLT), mean corpuscular volume (MCV), mean corpuscular hemoglobin (MCH), mean corpuscular hemoglobin concentration (MCH), red cell distribution width (RDW), and mean platelet volume (MPV). In parallel, additional blood analysis provided status of electrolytes (concentration of Na^+^, Ca^2+^, and Cl^−^), metabolites (glucose and lactate concentration), and blood (hemoglobin, hematocrit) (ABL815 Flex blood gas analyzer, Radiometer, Denmark). Here, blood after cardiac puncture was sampled in capillaries (safeCLINITUBES, Radiometer, Denmark) and stored at room temperature (< 2 h) until analysis.

### 2.8 Necropsy and histology

Directly after cardiac puncture, a routine dissection and visual inspection of the organs in the thoracic and abdominal cavity was performed for all mice. Thereafter, the stomach, duodenum, jejunum, liver, and kidneys were immediately removed and fixed in formaldehyde solution (4%). The small intestine was flushed with sodium chloride (0.9% Braun, Germany) and prepared in an improved Swiss-roll technique (36). After at least 3 days of formaldehyde fixation, tissues were trimmed according to the RITA Guidelines (37–39), dehydrated (Shandon Hypercenter, XP, Microm GmbH, Germany), and subsequently embedded in paraffin (Histoplast, Thermo Scientific, UK). Sections (2- to 3-μm-thick, microtome Leica RM 2245, Germany) were deparaffinized in xylene and H&E stained according to standard protocols. In order to analyze a representative sample of 14 individuals, from each cage, the organs of one mouse were randomly chosen for histopathological analysis (resulting in seven male and seven female mice assessed). A trained veterinary pathologist (SG) performed blinded evaluation (Axioskop 40, Carl Zeiss AG, Germany) and scoring of the sections (stomach, intestine). The kidneys and liver were evaluated for overall pathological findings. The scoring of the small intestine and stomach considered three general criteria according to a published protocol [cf. Table 1 of (40)]: (1) inflammatory cells, (2) intestinal architecture, and (3) degree of ulceration, if present.

The criterion (1) inflammatory cells were subdivided into evaluation of severity and the maximal extent of the inflammatory cells regarding the histologic layers of the mucosa and each graded from 0 to 4, resulting in a maximum possible score of 8. The criterion (2) intestinal architecture was subdivided in evaluation of the epithelial and the mucosal layer and each graded from 0 to 4, resulting in a maximum possible score of 8. The criterion (3) degree of ulceration included score values from 0 to 3, and the area involving ulceration included score values from 0 to 4. Score values for all criteria per organ were added up, resulting in an overall maximum score of 15 for the stomach and 23 for the intestine. Representative images of histological sections were taken on an Axioskop 40 microscope utilizing the software ZEN (Version 3.5 Blue Edition, Carl Zeiss AG, Germany).

### 2.9 Statistical analysis

Animal-individual data were recorded for carprofen plasma levels, body weight, clinical score, grooming activity, Irwin test parameters, mouse grimace scale, body temperature, tail immersion test, hematology, and histology, whereas data for food and water consumption, nesting, burrowing, and wheel running activity were recorded on cage level. Results are presented as mean and individual data points (plasma concentration-time curves); mean ± standard deviation (carprofen intake, wheel running, burrowing, tail withdrawal latency, body temperature, body weight, blood parameters, fluid and food consumption); median and individual data points (sum score Irwin test); or median and range (nest score, grooming score). To test for normal distribution of data, D'Agostino and Pearson test, Anderson–Darling test, Shapiro–Wilk test, and Kolmogorov–Smirnov test were conducted. Comparisons of two groups were performed by unpaired two-tailed Student's *t*-test (blood parameters) or Wilcoxon matched-pairs signed rank test [time to reach score 5 (nesting), body temperature]. Multiple comparisons were analyzed by repeated measures one-way Friedman-ANOVA (burrowing, nesting), followed by Dunn's multiple comparisons test, or Šídák's multiple comparisons test (food, water), and/or Kruskal–Wallis test [time to reach score 5 (grooming), sum score Irwin test, tail withdrawal latency], or two-way ANOVA including repeated measures and Šídák's multiple comparisons (wheel running). GraphPad Prism 9 [Version 9.3.1, GraphPad Software, USA, GraphPad Prism (RRID:SCR_002798)] was used for all statistical analyses. A *p*-value < 0.05 was considered statistically significant. Prior to experiments, power analysis was performed to calculate group size (*n* = 6) according to previously published data for latency to tail withdrawal in the hot water tail immersion test as primary read-out parameter (41).

## 3 Results

### 3.1 Favorable half-life of carprofen after s.c. injection and stable plasma concentrations during oral intake via d.w.

**Figure 2:**
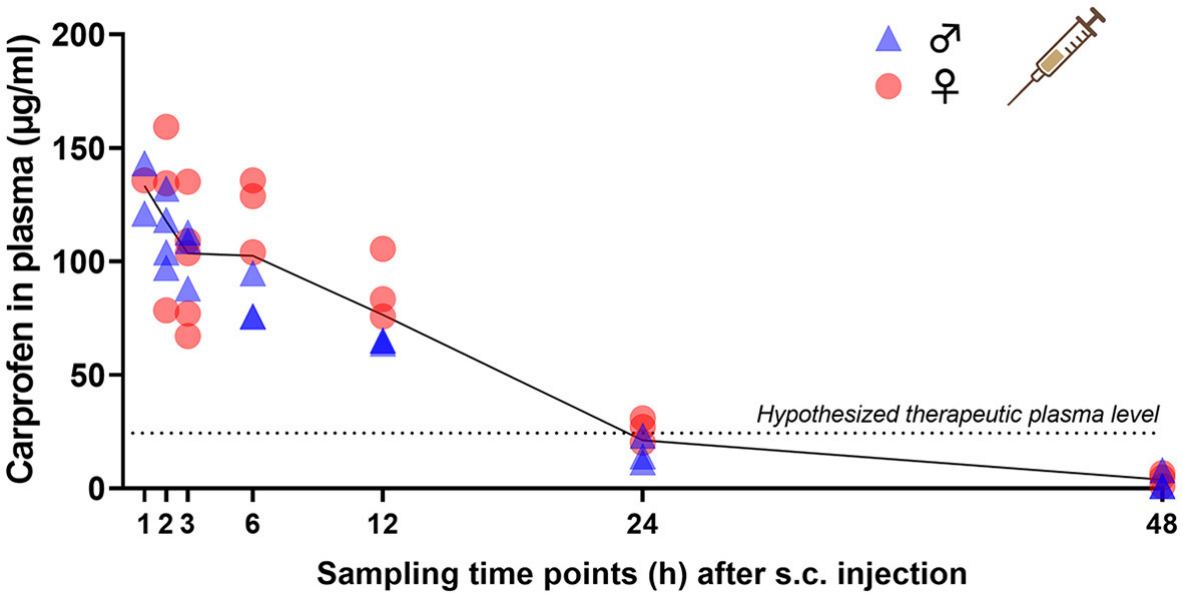
Plasma concentration-time curve following single s.c. injection of 20 mg/kg carprofen. Individual data points (*n* = 6, 3 male and 3 female per time point; exceptions: s.c. 1 h *n* = 2 male, 1 female; s.c. 2 h *n* = 4 male, 3 female; s.c. 3 h *n* = 3 male, 5 female) and mean of all animals per time point (black line) are presented. Assumed minimal therapeutic plasma level (dotted line) is displayed according to (7).

**Figure 3:**
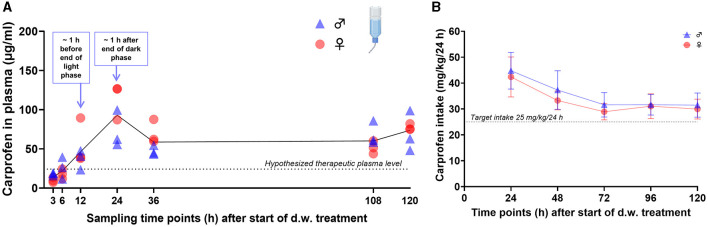
Carprofen plasma levels and dose intake during oral self-administration. **(A)** Plasma concentration-time curve during oral intake of carprofen (intended dose: 25 mg/kg/24 h) via drinking water (d.w.) over five consecutive days. Individual data points (*n* = 6, 3 male and 3 female per time point) and mean of all animals per time point (black line) are presented. Assumed minimal therapeutic plasma level (dotted line) is displayed according to (7). **(B)** Carprofen intake (mg/kg) is displayed for 24 h intervals after the start of oral treatment and is above target intake dose of 25 mg/kg/24 h (dotted line). Dose intake was calculated by consumption and concentration of carprofen-medicated drinking water.

To generate PK profiles of carprofen in mice, tail vein blood was collected at 1, 2, 3, 6, 12, 24, and 48 h after s.c. injection and at 3, 6, 12, 24, 36, 108, and 120 h after the start of oral self-administration. Single s.c. administration of 20 mg/kg carprofen resulted in maximum plasma concentrations of 133.4 ± 11.3 μg/ml after 1 h and an elimination half-life of 8.52 h (Figure 2). Plasma concentrations were above an assumed therapeutically useful level of 24.3 μg/ml, representing the *in vitro* canine whole blood assay IC80 value for COX-2 inhibition (10), for up to 24 h after injection. Oral self-administration of carprofen (25 mg/kg/24 h) started in the morning of the light phase and resulted in a steady state < 24 h after the start of the treatment with a plasma level of around 60 μg/ml (Figure 3A). Maximum plasma concentrations of 93.0 ± 30.6 μg/ml were observed after 24 h. Carprofen-medicated water was well-accepted and resulted in increased drinking water intake compared to BL2, especially in the first 24 h after the start of treatment for both sexes (*p* < 0.0001) (Supplementary Figure 5A). Additionally, male mice showed increased consumption after 48 h (*p* = 0.0008) and 120 h (*p* = 0.0439). During the first 24 h, male mice consumed 79% and female mice 86% more water compared to BL2 measurements (Supplementary Figure 5A). The increased water intake resulted in an ingested carprofen dose of 43.6 ± 7.2 mg/kg (males 44.8 ± 7.1 mg/kg, females 42.4 ± 7.7 mg/kg) within the first 24 h, and intake of the target dose (25 mg/kg/day) was almost reached at 12 h after the start of the treatment (Supplementary Figure 2A). From 72 h to the end of the experiment, water intake was stable for both sexes and resulted in dose intake of approximately 30 mg/kg/24 h (Figure 3B). Regarding circadian rhythm, the larger amount of carprofen was ingested during the dark phase (Supplementary Figure 2B). During the light phase, mice consumed between 49% (0–24 h) and 35% (96–120 h) of total carprofen intake. No obvious differences between female and male mice were noted for plasma levels resulting from both routes of administration.

### 3.2 Carprofen is well-tolerated at single and prolonged high-dose treatment

**Figure 4:**
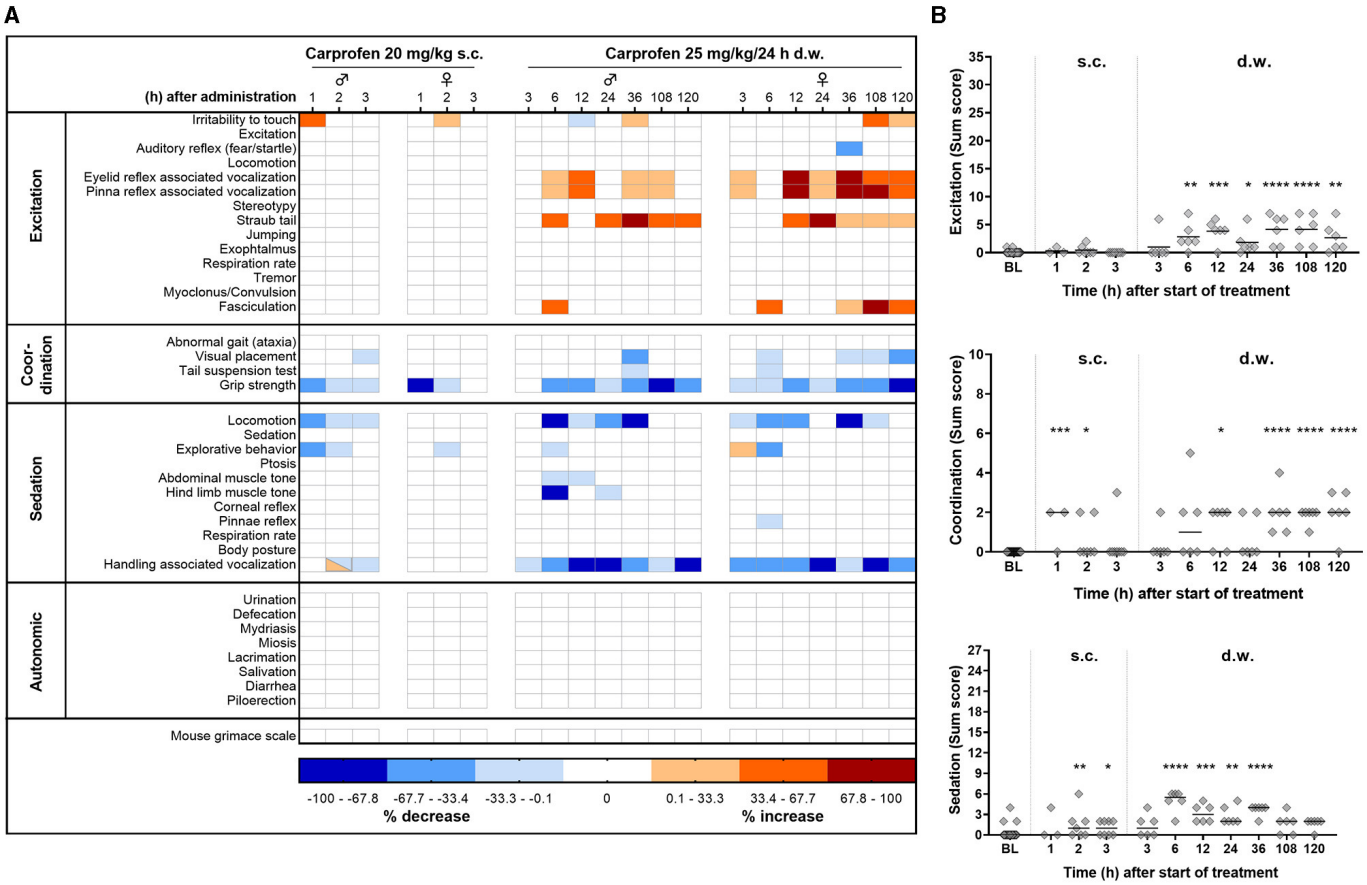
Tolerability of carprofen assessed by modified Irwin test. **(A)** Side effects of carprofen on behavioral parameters are displayed as heat map. Per time point and sex, three mice were investigated (exceptions: s.c. 1 h *n* = 2 male, 1 female; s.c. 2 h *n* = 4 male, 3 female; s.c. 3 h *n* = 3 male, 5 female). Increase or decrease is presented as a percentage of the change in the total number of animals per sex and timepoint. **(B)** Kruskal–Wallis test followed by Dunn's multiple comparisons test show significantly higher sum score values compared to baseline in the categories excitation, coordination, and sedation (*p < 0.05; **p < 0.01; ***p < 0.001; ****p < 0.0001).

Tolerability and potential side effects were evaluated by a modified Irwin test protocol including a large variety of read-out parameters (Supplementary Table 1). Investigated parameters were categorized into excitation, coordination, sedation, and autonomic symptoms and displayed in a heat map, including also the mouse grimace scale (Figure 4A). After s.c. injection, both sexes showed a slight decrease in grip strength, whereas male mice also showed slightly reduced locomotion/exploration. During oral intake, in both sexes, excitation was observed, represented by increased vocalization during reflex testing (eyelid, pinna) and by increased tail elevation (straub-like). However, handling-induced vocalization was reduced. Coordination, visual placement, tail suspension test, and grip strength were reduced in some individuals. Pointing to an activity-reducing effect, decreased locomotor activity was observed. For the categories excitation, coordination, and sedation, a sum score for each individual animal and time point is presented in Figure 4B. After s.c. injection, increased score values for coordination and sedation were observed (coordination: 1 h, *p* = 0.0003, 2 h, *p* = 0.0408; sedation: 2 h, *p* = 0.0095, 3 h, *p* = 0.0262), whereas during d.w. treatment significant changes compared to baseline in all three categories were present. However, deviations in score values were mainly expressed in a low grade of Irwin score. Live scoring of the mouse grimace scale (12) was performed as part of the Irwin test procedure (Figure 4A) in the home cage and did not reveal increased score values at any time point.

**Figure 5:**
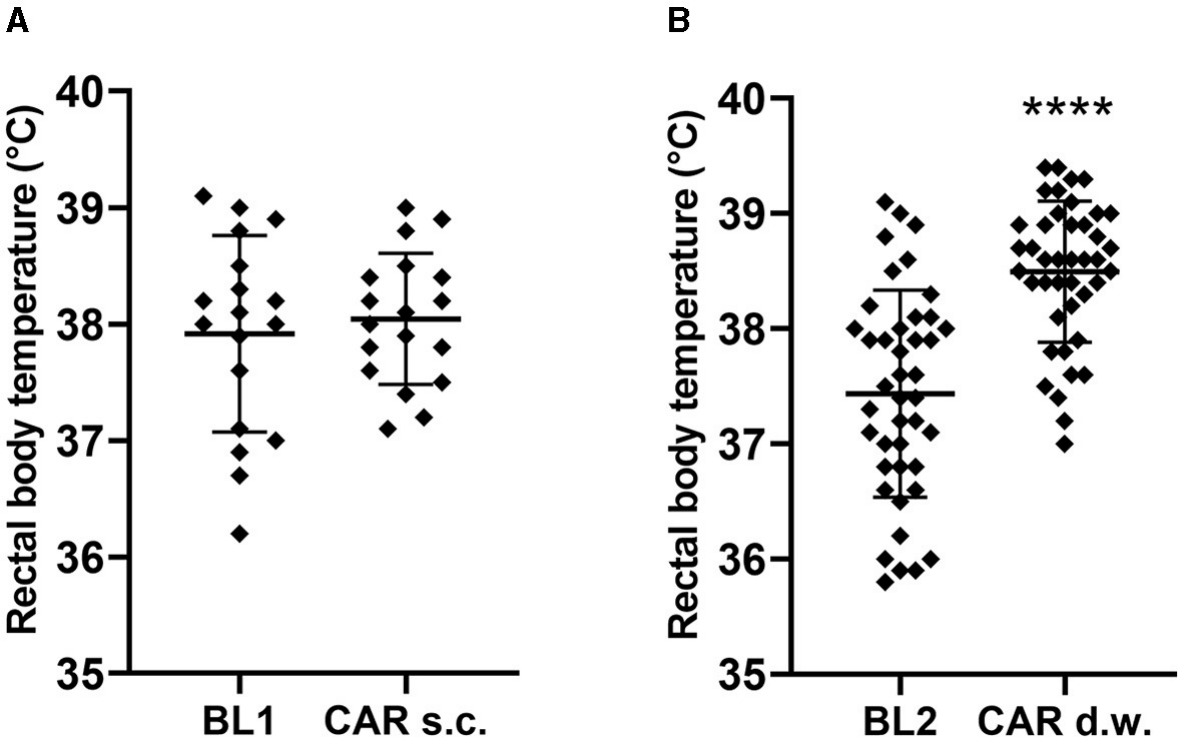
Carprofen intake via d.w. influences body temperature. Data from all measurements after s.c. carprofen treatment **(A)** and intake via d.w. **(B)** are shown. Wilcoxon test compared paired data after s.c. (*n* = 18; nine male, nine female) and d.w. (*n* = 42; 21 male, 21 female) treatment and shows increased body temperature during d.w. treatment, but not after s.c. injection of carprofen compared to individual baseline (****p < 0.0001).

For rectal body temperature, no sex differences were observed comparing individual baselines (BL1 male vs. female, *p* = 0.1619; BL2 male vs. female, *p* = 0.1676). However, despite habituation procedures, a significant difference between the first and second baseline measurements was present for both sexes (male: BL1 38.13 ± 0.93°C, BL2 37.63 ± 1.01°C, p = 0.0295; female: BL1 37.80 ± 0.50°C, BL2 37.24 ± 0.67°C, *p* = 0.0004; Supplementary Figure 6). Therefore, corresponding BL1 and BL2 data were used for statistical analysis of carprofen's potential impact on body temperature (Figure 5). After s.c. carprofen injection, no differences in body temperature compared to the corresponding baseline measurement were found (BL1: 37.92 ± 0.84°C, s.c. 38.04 ± 0.56°C, *p* = 0.9716) (Figure 5A), but a significantly increased body temperature was observed during d.w. treatment (BL2: 37.44 ± 0.90°C, d.w. 38.50 ± 0.61°C, *p* < 0.0001) (Figure 5B).

A clinical score (Supplementary Figure 1A) was assessed two times per week during baseline phases and daily during s.c. (until day 2 after injection) and d.w. treatment, including activity, general condition, behavior, body posture, and body weight. In only two female and one male mice, minor and transient increases in clinical score were observed during carprofen treatment (Supplementary Figure 1B). No signs of skin irritation were observed after s.c. injection. The body weight during experiments was stable in both sexes (Supplementary Figure 4), but female mice exhibited a slight decrease in food consumption 120 h after the start of carprofen administration via d.w. (*p* = 0.0086) (Supplementary Figure 5B).

### 3.3 Impact of prolonged treatment on home-cage behavior

**Figure 6:**
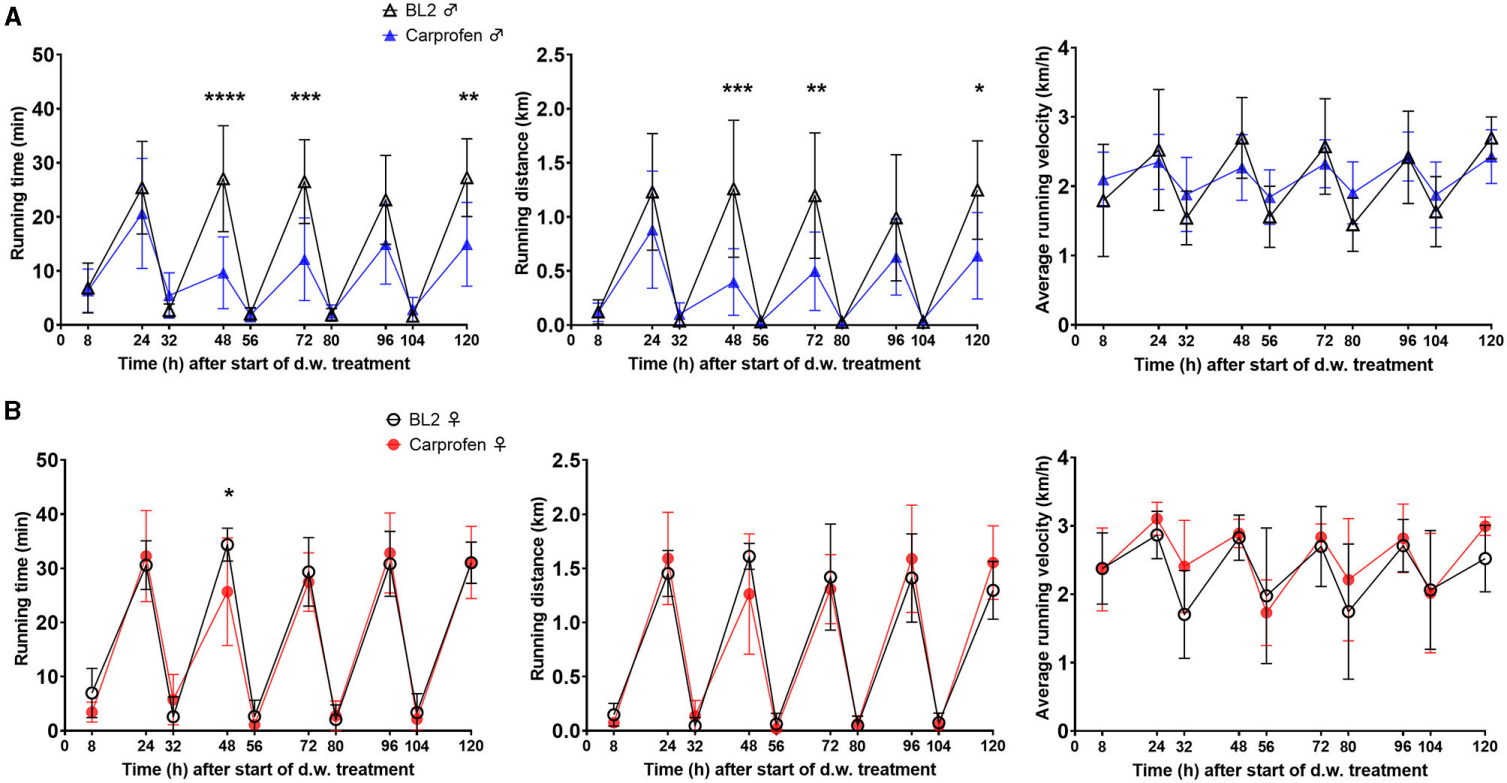
Carprofen treatment via d.w. is associated with decreased wheel running activity in male mice. Running time (min), running distance (km), and average running velocity (km/h) during the “night” phase (4:00–6:00 p.m. to 8:00–9:00 a.m.) are shown normalized to total time (h) during read-out intervals for **(A)** male and **(B)** female mice. Data are shown as mean ± SD (*n* = 7 cages per sex, three mice/cage). Two-way ANOVA and Šídák's multiple comparisons test reveal a decrease in wheel running time and distance in male mice under carprofen treatment (*p < 0.05; **p < 0.01; ***p < 0.001; ****p < 0.0001).

During the five consecutive days of carprofen administration via d.w., wheel running activity was measured to detect potential influence of analgesic treatment on voluntary exercise. Male mice showed a significant decrease both in running time overnight (min) after 48 h (−70%; *p* < 0.0001), 72 h (−55%; *p* = 0.0002), and 120 h (−49%; *p* = 0.0048) and running distance overnight (km) after 48 h (−75%; *p* = 0.0001), 72 h (−55%; *p* = 0.0021), and 120 h (−52%; *p* = 0.0315) compared to respective time points during BL2 (Figure 6A). Female mice displayed comparable wheel running activity to BL2, except for a decrease in running time overnight (min) after 48 h (−25%; *p* = 0.0168) (Figure 6B). For both sexes, running time, distance, and velocity within the light phase were not affected by analgesic treatment.

**Figure 7:**
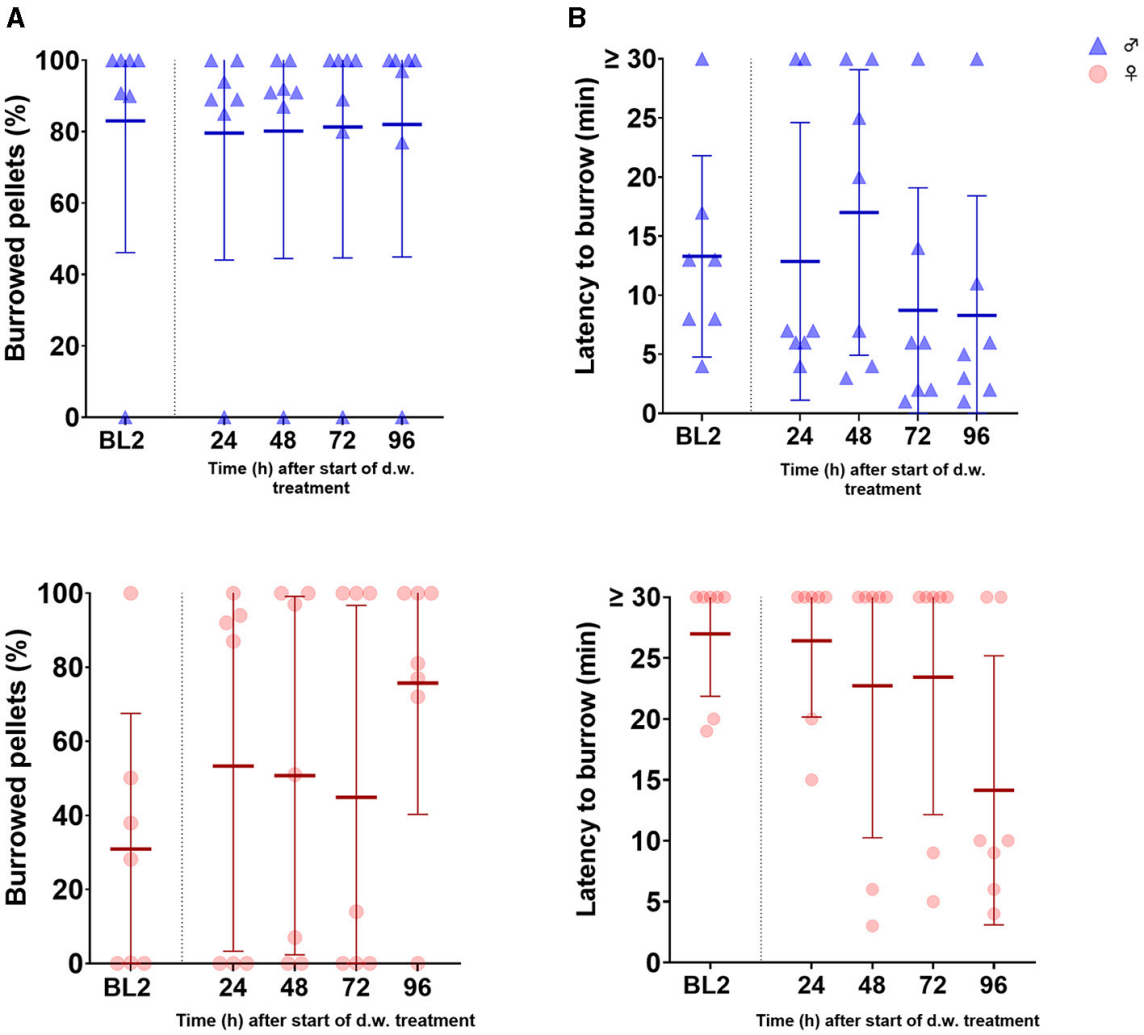
Burrowing behavior is not influenced by carprofen treatment via d.w. **(A)** Latency time to start of burrowing behavior after placing the burrowing tube in the cage for male **(top)** and female **(bottom)** mice, *n* = 7 cages per sex, three mice/cage. Data are shown as mean ± SD. Friedman-ANOVA and Dunn's multiple comparisons test did not reveal different burrowing activity in male or female mice during carprofen treatment vs. BL2. **(B)** No changes in the volume of burrowed food pellets [in percentage after 2 h for male **(top)** and female **(bottom)** mice, *n* = 7 cages per sex, three mice/cage] were observed.

Burrowing performance was assessed every day (in 24-h intervals) after the start of carprofen administration via d.w. During the burrowing time of 2 h, male mice burrowed continuously stable amounts of pellets both at baseline phase and during carprofen treatment. For female mice, we observed a somewhat higher fluctuation in burrowing activity (latency and amount burrowed) both during baseline phases and during carprofen administration (Figures 7A, B). However, no significant differences in burrowing latency or amount burrowed were found.

**Figure 8:**
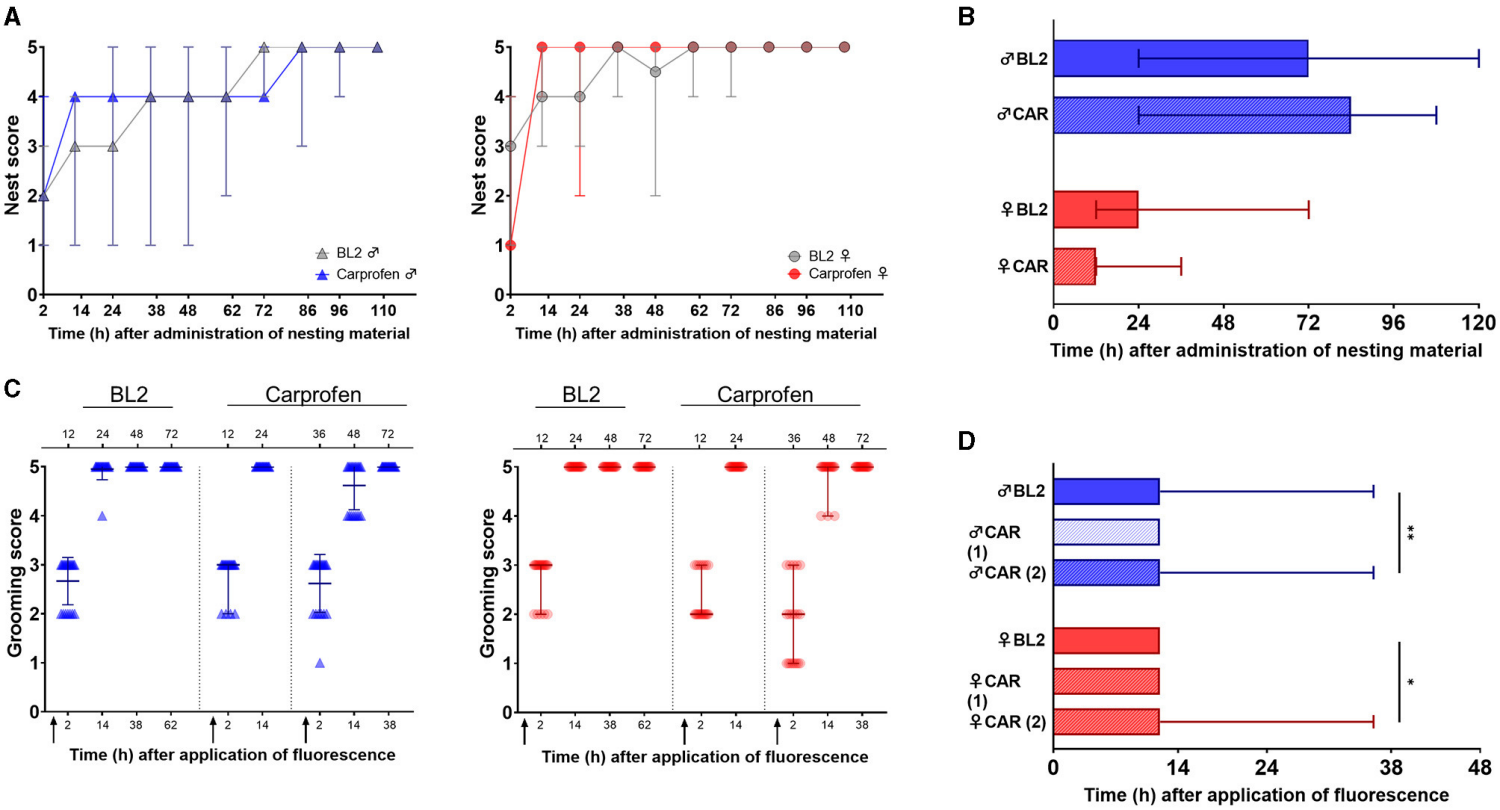
Nesting behavior remains unaffected, and grooming activity is slightly prolonged during carprofen treatment via d.w. **(A)** Nest scores after administration of nesting material for male **(left)** and female **(right)** mice. Nesting material was provided 10 h after the start of d.w. treatment. Data are presented as median and range (*n* = 7 cages per sex, three mice/cage). Friedman's test and Dunn's multiple comparisons test did not reveal significant differences at any time point. **(B)** Time (h) after administration of nesting material until nest score 5 was achieved is displayed. Data are presented as median and range (*n* = 7 cages per sex, three mice/cage). Wilcoxon test did not reveal altered nest building time during d.w. treatment. **(C)** Grooming scores of individual animals are shown for male **(left)** and female **(right)** mice as median and range (*n* = 21 per sex). Fluorescence suspension was applied 10 h after the start of d.w. treatment to mouse skin. Upper *x*-axis indicates time points during BL2 and after the start of d.w. treatment (Carprofen). Lower *x*-axis shows time points after administration of fluorescence suspension. Arrows indicate the administration of fluorescence. **(D)** Time (h) after application of fluorescence to achieve grooming score 5 is shown. Data are presented as median and range (*n* = 21 mice per sex). Kruskal–Wallis test followed by Dunn's multiple comparisons test show significantly prolonged time to achieve score of 5 for male (^**^p = 0.0044) and female (^*^p = 0.04488) mice under carprofen d.w. treatment vs. BL2 in the second trial.

Nesting behavior was assessed over five consecutive days twice daily using an established nest scoring protocol (6). Score values under carprofen treatment developed comparable to median of BL 2 in mice of both sexes (Figure 8A). Time to achieve nest score 5 was unchanged for male and female mice under carprofen medication (Figure 8B).

Grooming activity was assessed at 2, 12, 36, and 60 h after the start of fluorescent application to the neck region according to an established scoring protocol (6). Under carprofen treatment, the oil was applied two times (10 and 34 h after the start of the treatment). Grooming activity, represented by score values, developed similarly under treatment compared to median of BL 2 (Figure 8C). However, a slightly prolonged time to achieve score of 5 was observed for male (*p* = 0.0044) (Figure 8D) and female mice (p = 0.0488) in the second trial.

### 3.4 No anti-nociceptive effect in the hot water tail immersion test

**Figure 9:**
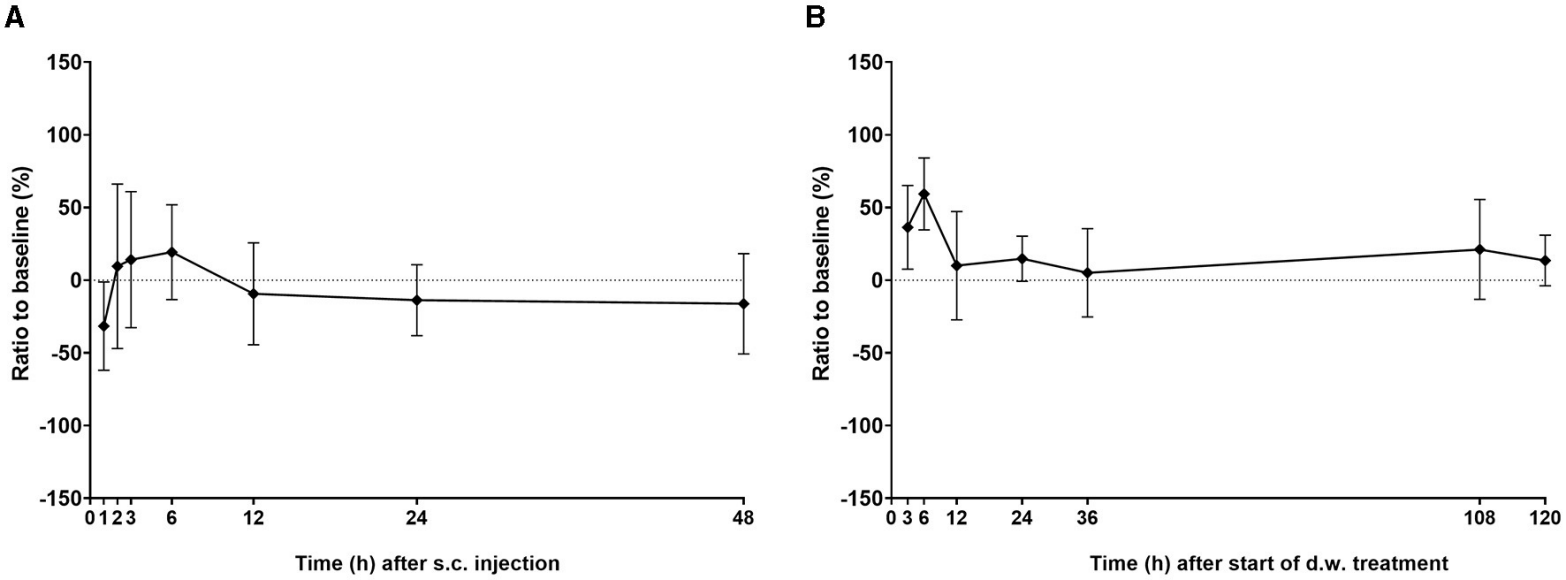
Anti-nociceptive efficacy of carprofen after s.c. injection and during treatment via d.w. Latency to tail withdrawal in the hot water tail immersion test at respective time points after **(A)** single s.c. injection and after **(B)** oral d.w. treatment. Latency to tail withdrawal is displayed as ratio to animal-individual BL (carprofen s.c./BL1; carprofen d.w./BL2). Latencies were calculated as the mean of the total three measures per animal and time point. Per time point, *n* = 6 mice (three male, three female) are shown (exceptions: s.c. 1 h *n* = 2 male, 1 female; s.c. 2 h *n* = 4 male, 3 female; s.c. 3 h *n* = 3 male, 5 female). Kruskal–Wallis test followed by multiple comparisons test did not reveal differences to baseline ratios at any time point after treatment.

Hot water tail immersion test was performed to test anti-nociceptive efficacy by latency to tail withdrawal from hot water. No statistically significant anti-nociceptive effects in the chosen test paradigm were found both after s.c. (Figure 9A) and d.w. administration (Figure 9B). Testing for a relationship of carprofen concentration in plasma and tail withdrawal latencies does not show statistical correlation (Supplementary Figure 3).

### 3.5 Comprehensive blood analysis and histological examination do not indicate adverse effects

**Figure 10:**
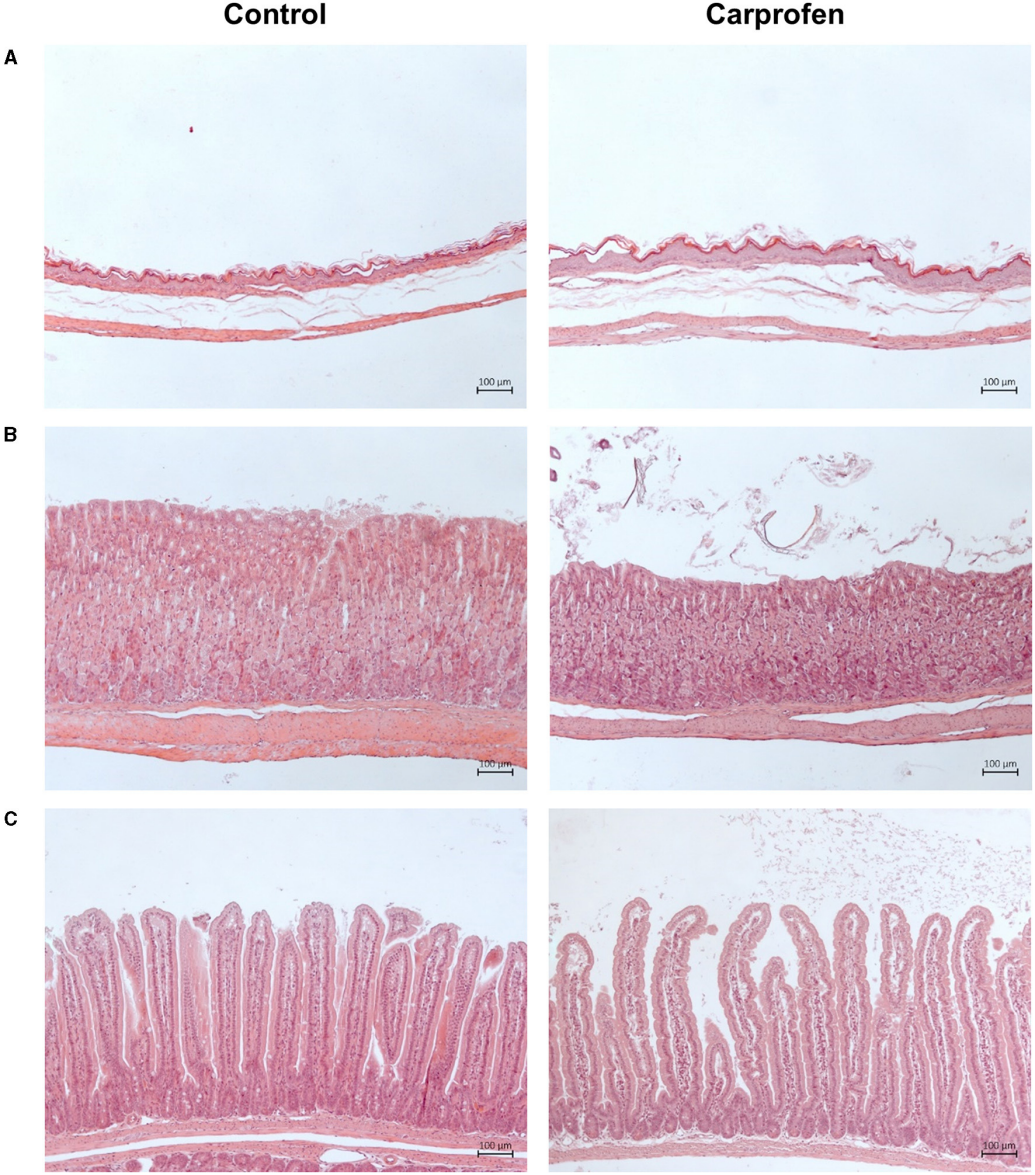
Representative hematoxylin–eosin staining of **(A)** forestomach **(B)** glandular stomach and **(C)** proximal part of jejunum of control animals **(left)** and after 5 days of carprofen d.w. treatment **(right)**. Carprofen has no adverse effects on histology of selected organs.

To assess potential side effects of continuous carprofen treatment for 5 days via d.w., organs of interest (stomach, duodenum, proximal part of jejunum, liver, and kidneys) were examined in detail by a veterinary pathologist. No tissue damages or signs of inflammation were observed, and rated histology score values [age-matched control: 0.30 ± 0.87 (range 0–3) *n* = 12; carprofen 0.14 ± 0.53 (range 0–2) *n* = 14; data not shown] of the stomach and small intestine were comparable between groups. Representative images of histologic stomach and jejunum sections from control and carprofen-treated mice are shown in Figure 10.

A standard blood profile after cardiac puncture was determined after 120 h of continuous carprofen d.w. treatment and compared to age-matched healthy control animals. Leukogram of white blood cells (WBC), lymphocytes (LYM), monocytes (MO), and granulocytes (GRA) as well as red blood cell (RBC) count including hemoglobin (HGB), hematocrit (HCT), platelets (PLT), mean corpuscular volume (MCV), mean corpuscular hemoglobin (MCH), mean corpuscular hemoglobin concentration (MCH), red cell distribution width (RDW), and mean platelet volume (MPV) is provided in Table 1. Unpaired two-tailed Student's *t*-test revealed lower LYM cell count (103/mm^3^) (control vs. carprofen, *p* = 0.0156), HGB (g/dl) (control vs. carprofen, *p* = 0.0019), HCT (%) (control vs. carprofen, *p* = 0.0022), and MCH (pg) (control vs. carprofen, *p* = 0.0003). Electrolyte and metabolite data did not indicate deviations between control and carprofen-treated animals (Table 1). However, significantly lower HGB and HCT in carprofen-treated mice were confirmed by measurement in a second system.

**Table 1:** Blood count and analysis of electrolytes and metabolites.

**Parameter**	**Control**	**Carprofen**	**Analyzer**
WBC (10^3^/mm^3^)	8.52 ± 3.41	6.68 ± 2.84	a
LYM (10^3^/mm^3^)	6.49 ± 2.38	**4.77** ±2.00^*^	a
MO (10^3^/mm^3^)	0.38 ± 0.22	0.36 ± 0.21	a
GRA (10^3^/mm^3^)	1.65 ± 0.90	1.54 ± 0.90	a
RBC (10^6^/mm^3^)	10.99 ± 1.28	9.89 ± 1.79	a
HGB (g/dl)	15.16 ± 1.72	**12.85** ±2.27^**^	a
	15.86 ± 0.81	**13.58** ±1.71^****^	b
HCT (%)	55.82 ± 6.07	**47.38** ±8.47^**^	a
	48.81 ± 1.48	**41.73** ±5.16^***^	b
PLT (10^3^/mm^3^)	862.50 ± 440.70	1,249.00 ± 553.80	a
MCV (fm^3^)	50.75 ± 2.53	**48.00** ±1.82^****^	a
MCH (pg)	13.83 ± 0.78	**13.02** ±0.58^****^	a
MCHC (g/dl)	27.17 ± 0.44	27.16 ± 0.84	a
RDW (%)	14.14 ± 0.45	14.60 ± 1.12	a
MPV (fm^3^)	5.77 ± 0.14	5.73 ± 0.35	a
cNa^+^ (mmol/L)	152.60 ± 3.00	152.50 ± 3.21	b
cCa^2+^ (mmol/L)	1.58 ± 0.03	1.62 ± 0.09	b
cCl^−^ (mmol/L)	117.00 ± 2.60	118.00 ± 3.20	b
cGlu (mmol/L)	12.64 ± 1.79	13.49 ± 4.25	b
cLac (mmol/L)	7.16 ± 2.05	6.79 ± 1.68	b

Blood count, electrolyte, and metabolite analysis of naïve, age-matched control mice (*n* = 6 male, 6 female) and carprofen-treated mice (*n* = 21 male, 21 female) was performed after final cardiac puncture. Leukogram of white blood cells (WBC), lymphocytes (LYM), monocytes (MO), and granulocytes (GRA) is displayed. Red blood cell (RBC) count includes hemoglobin (HGB), hematocrit (HCT), platelets (PLT), mean corpuscular volume (MCV), mean corpuscular hemoglobin (MCH), mean corpuscular hemoglobin concentration (MCHC), red cell distribution width (RDW), and mean platelet volume (MPV). Concentrations of electrolytes, glucose, and lactate are shown.

Values are presented as mean ± SD.

Unpaired two-tailed Student's *t*-test was used to test for differences between control and carprofen-treated mice: ^*^p < 0.05, ^**^p < 0.01, ^***^p < 0.001, ^****^p < 0.0001.

The alphabet “a” indicates scil Vet abc, scil animal care company GmbH, Germany.

The alphabet “b” indicates ABL815 Flex blood gas analyzer, Radiometer, Denmark.

## 4 Discussion

As basis for evidence-based refinement of pain therapy using carprofen, this study determined its PK, tolerability, as well as impact on cage-side behaviors in healthy male and female C57BL/6J mice at highest recommended doses. Following a single s.c. injection of 20 mg/kg, C_max_ was reached before 1 h, and an elimination half-life (*t*_1/2_) of 8.52 h was determined. Plasma levels were found to be well-above an estimated therapeutic threshold up to 24 h. Carprofen-medicated d.w. (target dose of 25 mg/kg/24 h) was well-accepted and resulted in a steady-state plasma concentration within 24 h, which was maintained throughout the treatment phase of 5 days. High-dose carprofen treatment was well-tolerated, with only minor and transient side effects observed in the Irwin test battery. No acute anti-nociceptive effect was detected using the hot water tail immersion test. The cage-side surrogate pain indicators were either not influenced (grimace scale, burrowing, nesting) or only mildly, in the form of slightly delayed grooming behavior. Nonetheless, high-dose carprofen via d.w. decreased wheel running activity, seen mainly in male mice, up to 70%. Hematological and histopathological examination did not indicate any pathological effects on organs.

### 4.1 Current dose recommendations, PK data, and analgesic efficacy

Carprofen is a non-steroidal anti-inflammatory drug (NSAID) that is commonly used in laboratory rodents to relieve pain and inflammation. It is approved for clinical use in veterinary patients in the EU (various species) and the US (dog) in multiple preparations, but the application for pain relief in rodents remains off-label. For mice, dose recommendations cover a range of approximately 2–20 mg/kg s.c. every 12–24 h, depending on the source used (22, 42, 43). *In vivo* therapeutic plasma levels in mice are still unknown to date. However, current literature often refers to an estimated therapeutic threshold above 24.3 μg/ml calculated from *in vitro* data using canine blood cells (corresponding to a carprofen concentration inhibiting 80% of COX-2 activity) (10). Our study shows that plasma levels remain above this estimated threshold for up to 24 h in male and female C57Bl/6J mice when 20 mg/kg are injected s.c. This is in line with recent observations made in another mouse strain, i.e., female CD1 mice (13). The authors report a *t*_1/2_ of 10.2 h following s.c. injection of 20 mg/kg, matching the *t*_1/2_ of 8.52 h determined in the present study (13). Surprisingly, a somewhat shorter *t*_1/2_ of 6.88 h was previously reported for a sustained-release formulation (5 mg/kg s.c.) in female CD1 mice (11). Altogether, the comparatively slow elimination and maintenance of presumed minimal therapeutic plasma levels for at least 12 h [10 mg/kg s.c., (13)] and up to 24 h (20 mg/kg s.c.), the present study and (13) suggest that an injection interval of ≥12 h is appropriate for achieving consistently relevant plasma levels with medium to high carprofen doses in mice of different strains and both sexes.

The non-invasive and stress-free nature of medication via d.w. makes this approach worthy of further evaluation, especially as carprofen is reported to be stable in d.w. and does not exert negative effects on consumption in C57BL/6 mice (4). Administration via the d.w. generally harbors a certain risk of unintentional loss of small quantities of water due to the movement of the bottles or leaks. To avoid this, we handled cages and bottles always with extra caution, and animal caretakers were also instructed to carefully check bottles for leaks. Occasionally, mouse play behavior with the drinking nipples may cause unwanted water spilling. We did not record this in a structured manner, but such behavior was not observed at any time throughout the study. Nest building against drinking nipples, which can trigger water leakage, was also not observed. D.w. consumption was stable during individual BL measurements and between BL1 and BL2 (Supplementary Figure 5A), indicating reliability of this method. With a medium dosage (10 mg/kg/d), others found mean plasma concentrations of up to 32 μg/ml between 24 and 72 h after the start of the treatment in female CD1 mice (5) and of above 20 μg/ml at 36 h after the start of the treatment in male C57BL/6J mice (4). In the present study, self-intake of a high dose (≥25 mg/kg/d) led to plasma levels of around 60 μg/ml (maximum mean of 93.0 μg/ml) from < 24 h onwards, which is well-above the estimated therapeutic threshold, and remaining stable for the 5 days of treatment, and importantly also during the light phase. This means that even with expected transient reduction in d.w. intake after anesthesia and surgery, estimated therapeutic plasma concentrations are likely to be maintained with high-dose carprofen via d.w. Whether these concentrations provide sufficient analgesia for post-operative pain remains to be further evaluated, as evidence-based data on clinical efficacy of carprofen via d.w. in mice are scarce.

Nevertheless, there is evidence that carprofen doses of 20–25 mg/kg given as single s.c. injection immediately after surgery effectively reduce post-operative pain after laparotomy when the mouse grimace scale is used as pain indicator (12). Otherwise, 30 mg/kg/24 h in d.w. had only minimal benefit after laparotomy in mice (6). After craniotomy, doses of 10 and 25 mg/kg, both s.c. and oral per d.w., also demonstrated a significant reduction in mouse grimace scale scores, where injected carprofen was more effective immediately after surgery, and oral carprofen treatment was more effective in female mice (44). However, related plasma concentrations were not determined in these studies. When a low dose (5 mg/kg s.c.) was applied, the majority of studies did not observe clear pain relief after surgery (45–48), while few others reported effective analgesia after surgical intervention (49). Although findings on clinically effective doses of carprofen as single analgesic are not fully conclusive, they suggest that in most cases, medium to high carprofen doses are required for effective pain management. Assuming that 20 mg/kg is effective and that the plasma concentrations achieved with this dose in our study clearly exceed the assumed minimum threshold (24.3 μg/ml), it can be assumed that the latter are by no means too high. In line with this assumption, McKenna et al. (13) conclude from their recent findings post ovariectomy in female CD1 mice that the minimum plasma concentrations might even be higher than 20–24 μg/ml.

Of note, exact analgesic dose and dosing regimen required to provide sufficient pain relief can also vary depending on multiple factors such as the mouse strain, sex, age, the experimental procedure, and the individual animal's health status. Moreover, carprofen's analgesic effect is mainly mediated through its anti-inflammatory mechanism of action and will therefore not address all levels of pain perception, suggesting that a combination with analgesics of a different mechanism of action might be a more promising approach for strengthening pain relief than further dose increases. For future studies, we consider it also promising to combine the administration routes evaluated in the present study. For example, as already done in a recent collaborative study based on the present findings (7), carprofen treatment could already start on the day prior to surgery by d.w. in terms of a non-invasive pre-emptive analgesia. Subsequently, in addition to continuous carprofen supply via the d.w., an additional single s.c. injection could be undertaken after surgery to cover up for the reduction in d.w. intake (and thereby carprofen dose) to be expected in the post-anesthetic phase. Together with the application of local anesthetics, this might be a promising, predominantly non-invasive, multimodal analgesic approach for surgeries of minor to moderate severity.

### 4.2 Anti-nociceptive efficacy

The hot water tail immersion test is a commonly used method to assess thermal nociception. It is based on the principle that exposure to a noxious thermal stimulus, such as hot water, will elicit a reflexive withdrawal response, which will be prolonged by analgesics impacting thermal nociception (38, 52). It is sensitive to centrally acting analgesics like opioids but has shown response to anti-inflammatory drugs as well (32, 33). We therefore expected to find at least a weak increase in tail withdrawal latency for high-dose carprofen. However, despite plasma levels clearly above the estimated therapeutic threshold, we could not find an anti-nociceptive effect for both routes of administration, and consequently no correlation between plasma levels and latency of tail withdrawal in the present study (Supplementary Figure 3). Here it is important to mention that not only the plasma level might be considered as reference for therapeutic efficacy, but also analgesic concentration in the tissue and enzyme binding/inhibition, both of which were not determined. Furthermore, the applied tail immersion test, although having been shown to respond to NSAIDs (50–52), is much more sensitive for detection of anti-nociceptive effects by opioids.

The type of restrainment during the test (38) and the number and interval of repetitions (26) are known to influence the outcome of the test. Moreover, potential influence of stress-induced analgesia by handling or restraining should be taken into consideration (56). To avoid this confounding effect in our study, mice were habituated extensively to all handling procedures, including bathing the tail, before the start of experiments. In addition, a sex difference in thermal nociception in mice has been reported (53), which was confirmed by our results, as males exhibited longer latencies during baseline measurements compared to females. In addition, the outcome can depend on water temperature. For example, the test successfully detected anti-nociceptive effects of the NSAIDs diclofenac, etodolac, and indomethacin in mice at a water temperature of about 52°C (54, 55), whereas ibuprofen treatment prolonged tail withdrawal latency only at 45°C but not at 50°C (52). As ibuprofen and carprofen are related substances, we cannot exclude a more pronounced anti-nociceptive effect at a lower water temperature. To the best of our knowledge, long-term studies on how repeated hot water tail immersion tests might affect the latency to tail withdrawal in mice have not been published. Therefore, a sham control group could have added information in this regard. However, we found distinctly higher variability in the outcome of this parameter than expected according to published data (41), suggesting an increase in group size to reduce variance might be the most important adaptation to be made for future studies. As our study design focused on PK and tolerability, we cannot make statements about carprofen's potential impact on other tests or parameters for nociception or pain, such as the formalin test, carrageenan edema test, telemetry data, or blood biomarkers. We consider it appropriate to include these more invasive tests in future studies in which we would like to build on the present results.

### 4.3 Tolerability profile

Overall, only minor side effects were observed by the Irwin test, hematology, and histopathology, suggesting a good tolerability of carprofen in the investigated dose in male and female C57BL/6J mice. Evaluation of potential side effects of analgesics is important as they might put extra burden to the animal but also impact outcome measures of the scientific question. For tolerability assessment, we here thoroughly monitored the animals over a drug administration period of 5 days, corresponding to a length of time that might be required for the management of post-operative pain. Apart from clinical scoring and body weight measurement, we included in our study design a modified Irwin test battery (23) assessing treatment impact on 38 behavioral and physiologic parameters, body temperature, as well as comprehensive hematologic and histopathologic evaluation. The Irwin test revealed only a minor impact on coordination and slight excitatory and sedative effects during prolonged treatment. Main changes were increase in reflex-associated vocalization, decrease in drip strength, decreased locomotion, and decreased handling-associated vocalization. In general, however, mice were in a very good overall condition, and side effects were so mild that they did not become obvious during clinical scoring.

Intake of carprofen via d.w. induced a rectal body temperature increase of around 1°C (37.44 ± 0.08998, BL2, 38.50 ± 0.6117, carprofen). Since we observed a significantly lower body temperature at BL2 compared to BL1, a statistical analysis of carprofen's impact was performed by comparing the drug treatment data with the time-correlated baseline data (cf. Figure 1). It is known from humans that NSAIDs like ibuprofen can attenuate the normal circadian decrease in body temperature during nighttime hours, probably by suppression of both prostaglandin and melatonin synthesis (53). Therefore, it is not surprising to find carprofen affecting the body temperature of mice as well. While the altered body temperature observed here did not seem to impact the animal's welfare, it can still be a confounder to be aware of when using body temperature as an indicator of post-operative pain. For instance, at 12–24 h after a laparotomy without analgesia in mice, a body temperature increase of 0.5°C was reported (49). Moreover, habituation to procedure of body temperature measurement might influence the outcome. During our experiments, mice were not habituated specifically to this procedure but to the necessary restrainment.

Studies about carprofen effects on hematologic parameters in mice are sparse. In our study, electrolytes and metabolites were not affected. Blood count after 5 days of oral treatment revealed reduced lymphocyte concentration and tendency toward reduced white blood cells compared with control mice. A literature search did not reveal any evidence of NSAID-induced reduction of lymphocytes or white blood cells on systemic level. We can therefore only speculate that the reduction may be an aspect of the immunomodulatory effect of carprofen. The findings should be addressed in more detail in future studies. In this regard, we consider it an important first step to reproduce the finding, perhaps even in another mouse strain, and to determine how long the lymphocytes are reduced or whether this is even quickly reversible. Proteomics could be used for a more comprehensive investigation of the potential effects on the inflammasome. If such studies confirm the relevance of the finding, it seems appropriate to include hematological analysis as standard in future investigations and, since lymphocytes and white blood cells are critically involved in immune defense against infections, to pay particular attention to, e.g., wound repair or potential post-surgical infections in carprofen-treated mice. We also found decreased HGB and HCT values, which are most likely a consequence of blood sampling during our experiments (54), as histopathology did not reveal any signs of gastrointestinal bleeding. Although carprofen, like other NSAIDs, might be associated with a risk of gastric ulceration in mice (42), the detailed histologic examination of a representative part of the experimental group (*n* = 14/42 mice, both sexes) did not suggest any adverse effect on the stomach, duodenum, proximal part of the jejunum, liver, or kidneys after treatment over five consecutive days (≥25 mg/kg/d). In line with this, Kendall et al. (14) recently found that repeated s.c. injections of 20 mg/kg carprofen for up to 7 days in female mice of another strain (CD1) were not associated with toxic renal or hepatic effects, and serum chemistry remained unchanged. They also did not find impact of carprofen treatment on liver enzymes, such as ALT and AST, which were not determined in the present study due to limited blood volume available. In a recently performed collaborative investigation on analgesia refinement for craniotomies in mice, mild erosive lesions in the stomach were observed in 22% of mice after 5 days of oral treatment with carprofen-medicated water (7). However, plasma concentration in these mice was about 2.5- to 6-fold higher than in the present study, indicating that the threshold for gastrointestinal side effects is associated with comparatively high carprofen tissue levels in mice.

A limitation of our study is that we cannot exclude the possibility that a single high-dose s.c. injection of carprofen causes gastrointestinal side effects. However, additional mice would have been required for histological examination directly after s.c. injection. The use of a cross-over design would also not have allowed to discriminate between acute effects and potential long-term effects of the previous 5-day treatment with carprofen via d.w. Nonetheless, in view of the fact that in the present study, there were no long-term effects in the gastrointestinal tract after a 5-day treatment via d.w. as well as after a 7-day treatment via s.c. injection in another study in female CD1 mice (14), we consider it unlikely that a single high dose would adversely affect the gastrointestinal tract.

For a multimodal analgesic approach, the dose of the individual drugs should ideally be adjusted in such a way that side effects are limited while effective pain relief is provided. In mice with a clinical pain background, the present study design could be used in the future (after certain adaptation) to evaluate and optimize multimodal approaches, such as the combination of carprofen with the opioid buprenorphine, in terms of dose-finding and adjustment.

### 4.4 Impact on behavioral pain indicators

Analgesics might inadvertently influence the outcome of cage-side behavioral surrogate pain indicators commonly used after surgical interventions, such as burrowing, nesting and grooming behavior, or wheel running activity (15), which might limit their validity during post-operative pain assessment. On the other hand, awareness of this influence would allow considering this in the assessment. As cage-side behavioral surrogate markers of pain are increasingly integrated in experimental studies, for conclusive interpretation of outcomes, their sensitivity to confounding effects should be evaluated.

Burrowing and nest building are described as non-invasive and sensitive indicators of pain, distress, and suffering in mice, as these parameters can reflect their wellbeing and motivation (55). Mice with pain or under stress tend to burrow less and show a decrease in nesting performance compared to healthy mice (31). It was shown that burrowing in carprofen-treated mice (5 mg/kg) started earlier after laparotomy compared to controls without pain medication (47), supporting its utility for detecting analgesic efficacy as well. Here, burrowing behavior remained uninfluenced by carprofen administration. To improve the study design for future trials, confirmation of completed habituation seems desirable. This could be achieved by prolonging the habituation phase and by interim data analysis to confirm the stability of outcome measures. Furthermore, burrowing tubes could be additionally offered during the recovery phase to maintain stable habituation.

Nesting behavior can detect pain up to 24 h after laparotomy by scoring of nesting consolidation (6). The same study established grooming activity as a pain indicator for up to 48 h following surgery. Here, nesting activity remained unchanged during carprofen administration via d.w. Whereas, nesting is to monitor without disturbance of the animals, grooming activity (as assessed in our study) requires additional restraining and handling of the animal to administer the fluorescent oily suspension and to score the grooming activity. In our study, we applied the fluorescent suspension two times during carprofen treatment and found slightly increased times to achieve the highest grooming score in the second trial in both sexes.

Wheel running activity as a potential measure of pain in mice is a natural behavior shown voluntarily that can be objectively measured and quantified (27). It was shown to be sensitive to detect distress in a mouse model of colitis (56), inflammatory (57), or neuropathic pain (58). After surgical removal of the mammary fat pad, wheel running activity was reported to be reduced at 24 h independent of saline or analgesic treatment with carprofen, buprenorphine, or both (45). The authors concluded that the used opioid, on the one hand, had a sedative effect associated with ~80% less running activity, whereas the applied carprofen dose of 5 mg/kg (s.c.) had no sufficient analgesic effect, being associated with a running activity decrease of ~60%. Our finding of decreased running time (up to 70%) and distance (mainly in male) mice under carprofen treatment via d.w. underlines that knowledge about analgesic influence on this outcome measure is highly relevant for data interpretation.

Of course, behavioral parameters can be influenced by a variety of factors other than analgesic treatment. In the context of the present study, particularly the blood sampling as the most invasive procedure should be noted. Inclusion of a control group undergoing blood sampling, behavioral tests, and tolerability testing, but not analgesic treatment might therefore also have added information for interpreting the impact on pain indicators, especially wheel running and grooming activity. To follow the 3R concept, we made efforts to limit the number of animals by using a baseline-controlled instead of a sham-controlled study design. To ensure generation of most meaningful results, extensive habituation and repeated baseline measurements were performed. Control animals for histology were age-matched and kept under the same housing conditions. Moreover, repeated examination of the same individuals can reduce data variance and allow for paired data analysis, which increases statistical power.

Other behavioral parameters, such as ultrasonic vocalization (USV), might have been of interest to investigate. While USV seems to be a valuable parameter for investigating social interaction in laboratory rats and mice (59), the literature indicates that USV recording in the context of pain is complex and strongly influenced by the experimental design, highly variable between individuals, and in some cases not present at all (15). Our intention was to focus on cage-side behavioral indicators that are already commonly used and easy to implement in daily routine in every laboratory, while USV recording clearly requires specialized knowledge and equipment. For these reasons, we did not integrate USV recording in the present study.

## 5 Conclusion

PK and tolerability profiles of high doses of carprofen in male and female C57BL/6J mice, both for s.c. and oral self-administration, are favorable. Importantly, estimated therapeutic plasma concentrations were clearly achieved and maintained, and only minor side effects and no histopathological findings were observed. A certain impact of carprofen on cage-side behavioral surrogate markers of pain needs to be taken into account if these parameters are to be used to assess pain intensity. Provided that d.w. consumption is monitored, oral self-intake of carprofen instead of parenteral injections in the post-operative phase might represent a refinement option. Taken together, we consider high-dose carprofen safe and a promising candidate, especially for multimodal analgesic approaches.

## Data Availability

The original contributions presented in the study are included in the article/Supplementary material, further inquiries can be directed to the corresponding author.
