# Supplementary material for: Subcutaneous and orally self-administered high-dose carprofen shows favorable pharmacokinetic and tolerability profiles in male and female C57BL/6J mice
Source: Front Vet Sci. 2024 Sep 20;11:1430726. doi: 10.3389/fvets.2024.1430726 (PMC11457584; doi:10.3389/fvets.2024.1430726)
Supplement: Supplementary file 1 [file Data_Sheet_1.docx]

Supplementary Material

# Supplementary Figure 1


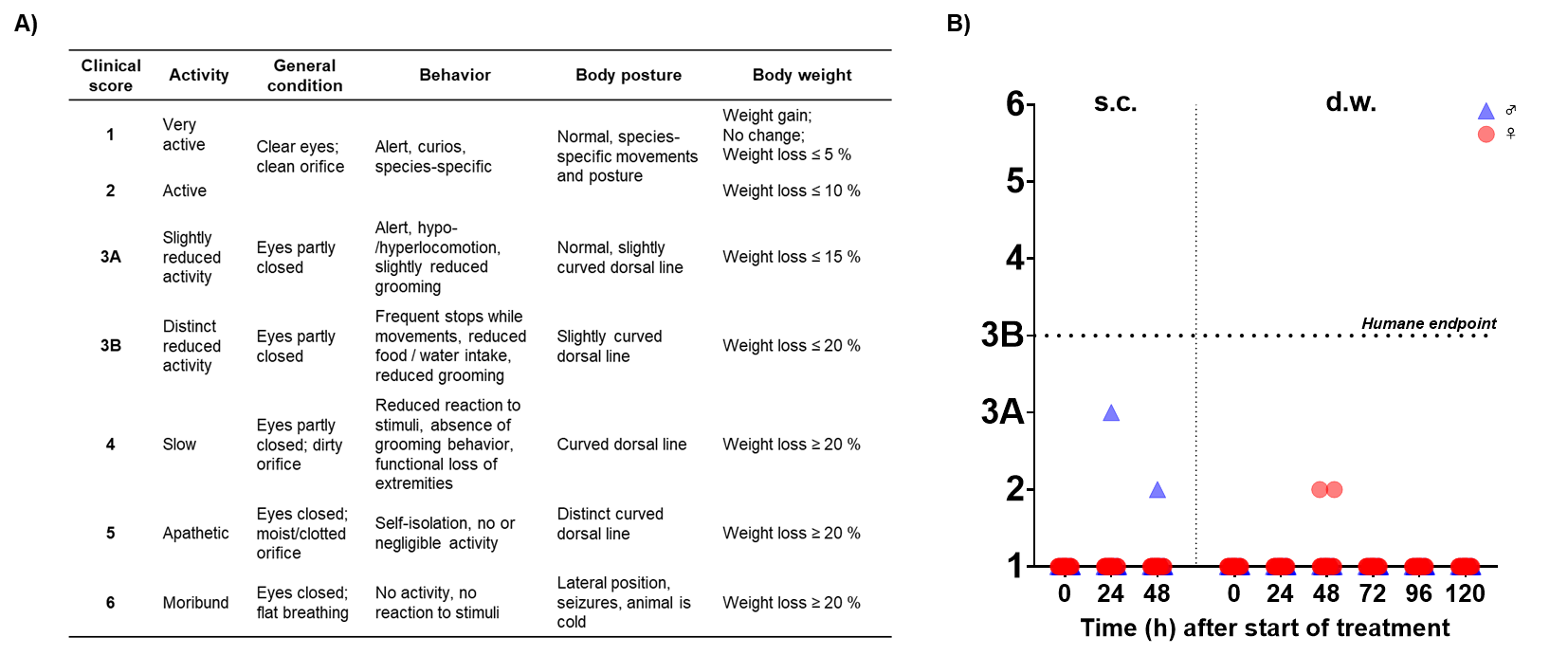


**Supplementary Figure 1.** **Clinical score. (A)** Clinical score was assessed daily during s.c. and d.w. treatment. **(B)** After s.c. injection, 1 male mouse showed an increased clinical score value after 24 and 48 h. During d.w. treatment, 2 female mice expressed an increased clinical score value at 48 h.

# Supplementary Figure 2


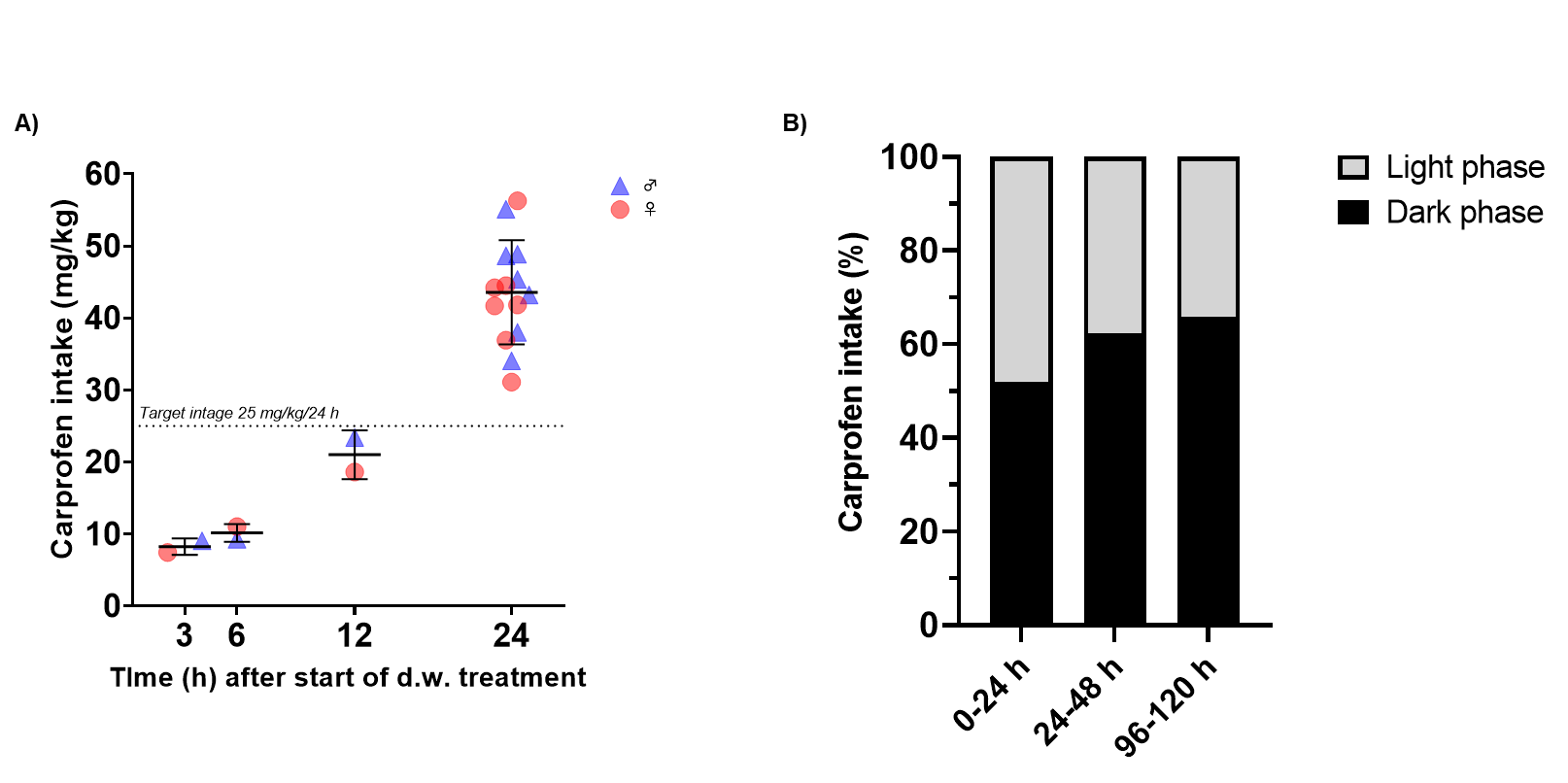


**Supplementary Figure 2. Carprofen intake during light and dark phase. (A)** Carprofen intake (mg/kg) within the first 24 h during d.w. treatment. Dose was calculated from individual water intake and concentration per cage and sex. **(B)** Carprofen intake (%) during the light and dark phase is displayed for 12 h intervals of total intake within 24 h (mean).

# Supplementary Figure 3

**Supplementary Figure 3. Carprofen plasma levels are not associated with latencies to tail withdrawal.** Latency difference of tail immersion test (%) shows no correlation (Spearman correlation) to level of carprofen in plasma (µg/ml). Simple linear regression (black line) describes the relationship between both variables graphically.

# Supplementary Figure 4


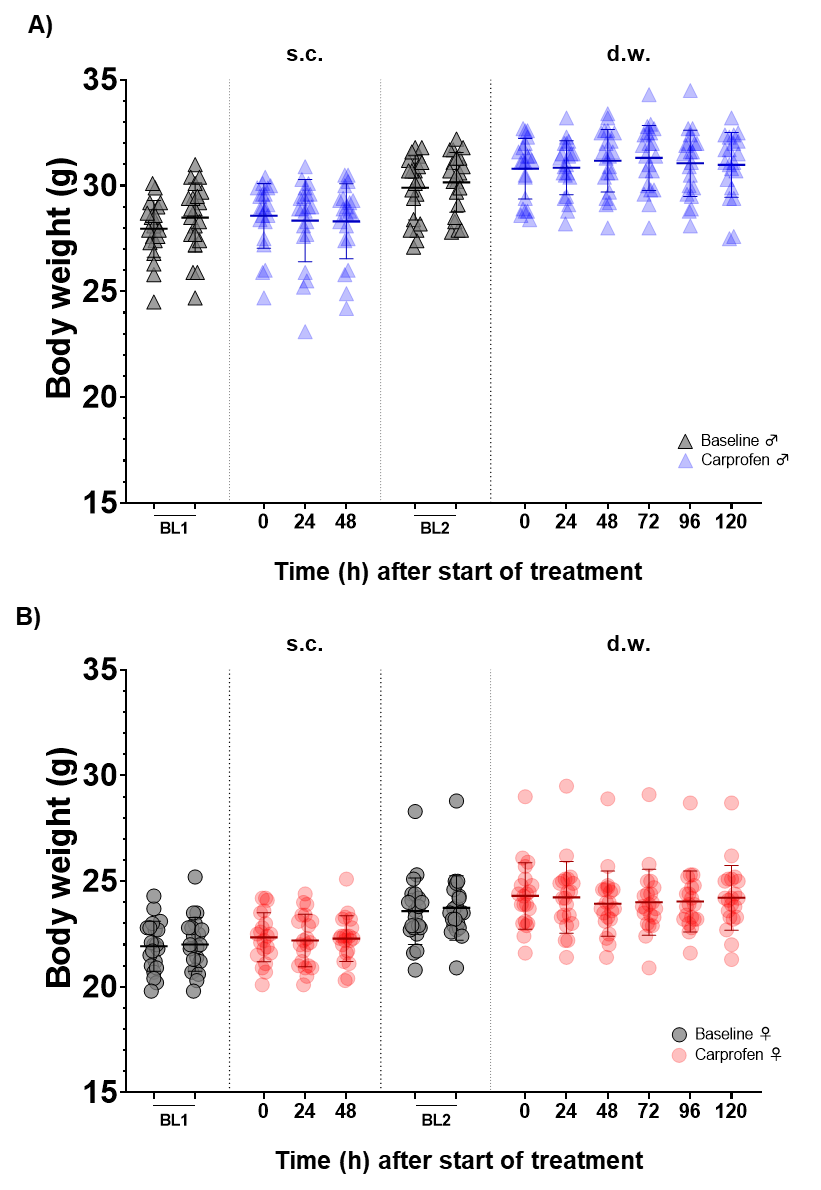


**Supplementary Figure 4. Development of body weight.** Daily body weight of **(A)** male and **(B)** female mice throughout the experimental course. Data points represent individual mice (n = 21 per sex), mean ± SD is shown. During baseline phases (BL1, BL2), mice were weighed 2x/week.

# Supplementary Figure 5

#

**Supplementary Figure 5. Fluid and food consumption.** **(A)** Consumption (g) of drinking water/24 h (BL1, BL2) and carprofen-medicated water (carprofen) is presented over 5 consecutive days for male (left) and female (right) mice. **(B)** Food consumption/24 h (g) during baseline and carprofen treatment is presented over 5 consecutive days for male (left) and female (right) mice. Data are shown as mean ± SD (n = 7 cages/sex; n = 3 mice/cage). Repeated measures one-way ANOVA followed by Šídák's multiple comparisons test was performed to test for differences between BL2 and carprofen treatment (****p<0.0001; ***p<0.001; **p<0.01; *p<0.05).

# Supplementary Figure 6


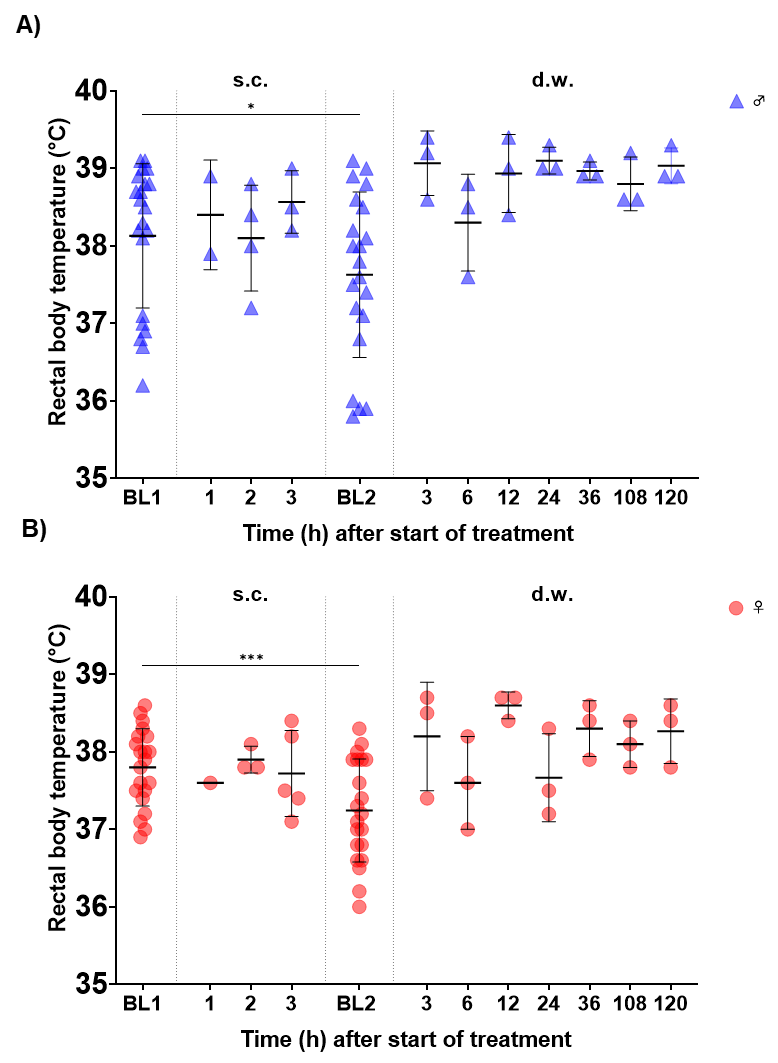


**Supplementary Figure 6.** Body temperature. Time course of rectal body temperature (°C) during baseline phases, as well as after s.c. and during d.w. treatment shown for **(A)** male and **(B)** female mice. Wilcoxon test shows differences between BL1 and BL2 for male (p=0.0295) and female mice (p=0.0004).

# Supplementary Figure 7

#
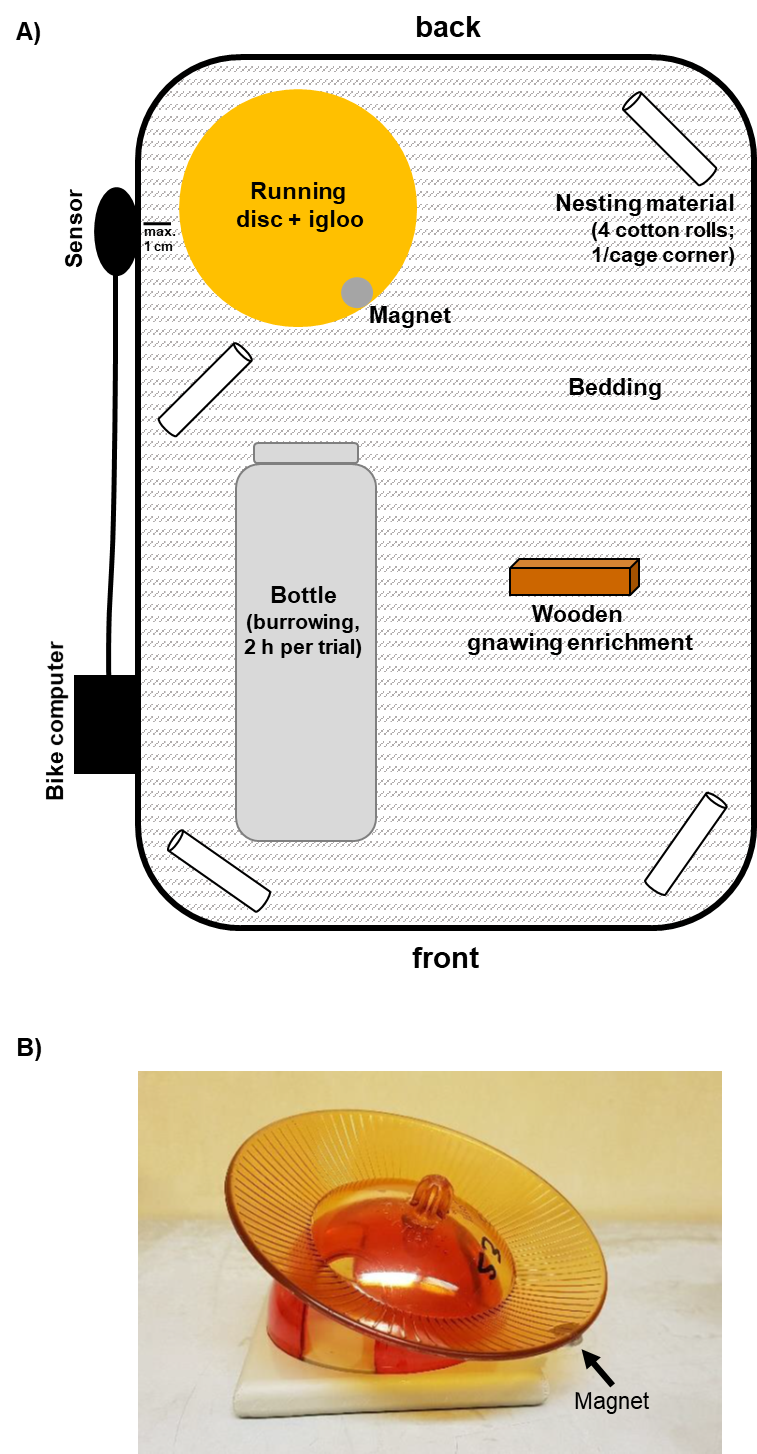


**Supplementary Figure 7. Experimental cage set-up. (A)** Schematic visualization of cage set-up in conventional Makrolon type III cages including running disc (mounted on igloo and pedestal), magnet, sensor, bike computer, nesting material, wooden enrichment , bedding and burrowing bottle (2 h per trial). **(B)** Photo of running disc and igloo on pedestal, arrow pointing at magnet.

# Supplementary Figure 8


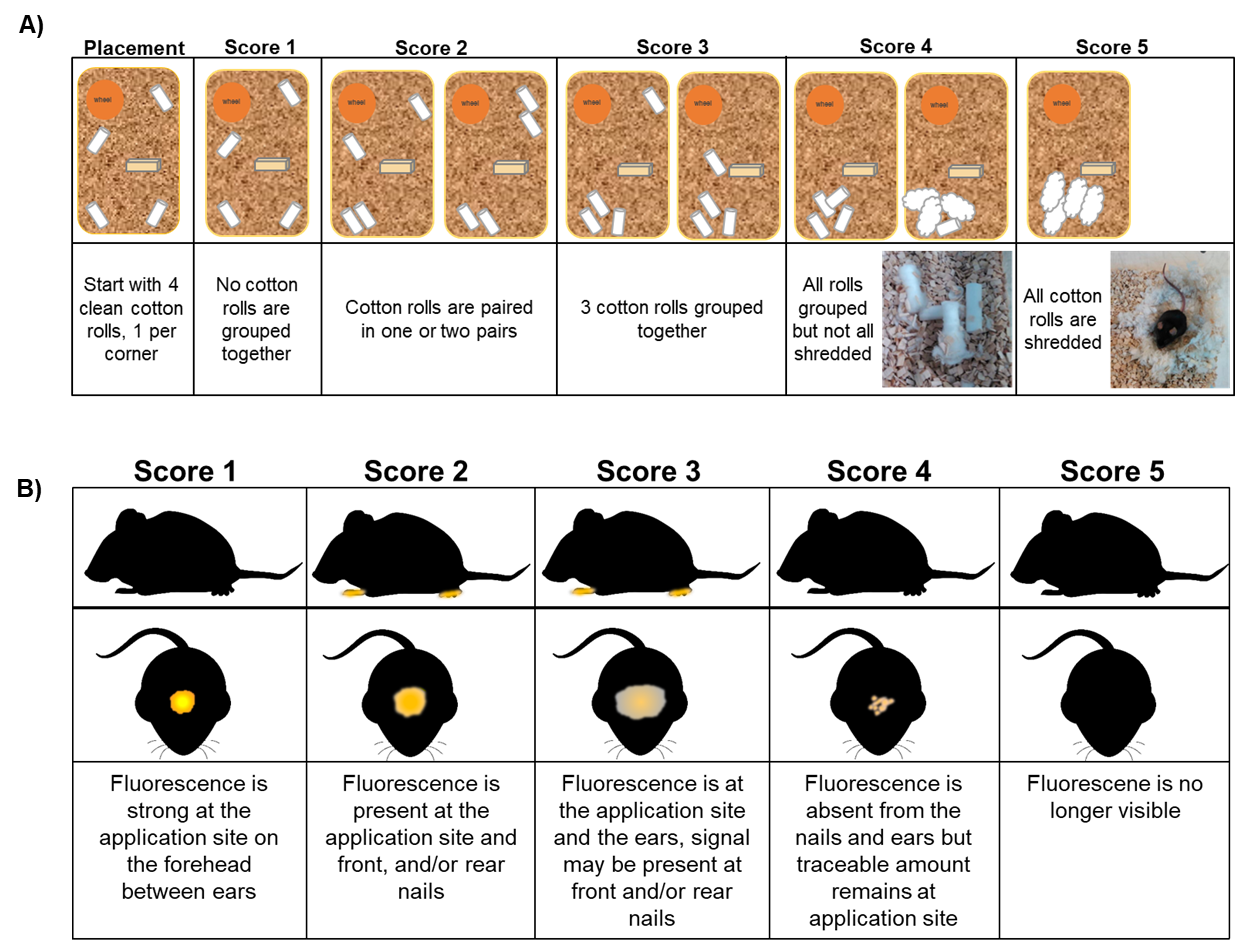


**Supplementary Figure 8. Nesting and grooming score.** **(A)** Nest consolidation score using cotton rolls and **(B)** grooming score (1).

1. Oliver VL, Thurston SE, Lofgren JL. Using Cageside Measures to Evaluate Analgesic Efficacy in Mice (Mus musculus) after Surgery. J Am Assoc Lab Anim Sci. 2018;57(2):186-201.

# Supplementary Table 1

**Supplementary Table 1.** Modified Irwin test parameters. Scores in bold are considered physiologic.

| Irwin test parameters |
| --- |
| I Observations without handling |
| Body position (- ; 0; +) |
| Locomotor activity (- ; 0; +) |
| Mouse grimace scale |
| - Orbital tightening (0; 1; 2) |
| - Nose/Cheek flattening (0; 1; 2) |
| - Ear changes (0; 1; 2) |
| - Whisker change (0; 1; 2) |
| Sedation (0; +) |
| Excitation (0; +) |
| Piloerection (0; 1; 2 ; 3; 4) |
| Ptosis (0; 1; 2 ; 3; 4) |
| Exophthalmos (0; +) |
| Unusual behavior (Free text, e.g. stereotypies, straub tail, circling…) |
| Myoclonus/Convulsion (- ; +) |
| Respiration rate (0; 1; 2 ; 3; 4) |
| Jumping (0; +) |
| Tremor (0; 1; 2 ; 3) |
| Auditory reflex (- ; 0; +) |
| II Observations open field |
| Ataxia (0; 1; 2; 3; 4) |
| Straub tail while walking (0; 1; 2 ; 3) |
| Locomotion (- ; 0; +) |
| Exploration (- ; 0; +) |
| III Observations with handling |
| Reactivity to touch (-1; 0; 1; 2 ; 3) |
| Aggressiveness towards handler (0; 1; 2) |
| Visual placement (-3; -2; -1; 0) |
| Tail suspension test (-1; 0; +1) |
| Grip strength (- ; 0; +) |
| Lacrimation (0; +) |
| Salivation (0; 1; 2) |
| Diarrhea (0; +) |
| Urination (0; +) |
| Defecation (Number of fecal boli) |
| Vocalization (Handling associated) (- ; +) |
| Abdominal tone (-2; -1; 0; +1; +2) |
| Limb tone (-2; -1; 0; 1; 2; 3) |
| Pinna and eyelid reflex (-2; -1; 0; +1) |
| Pupil size (Miosis/Mydriasis) (- ; 0; +) |
| Body temperature (Rectal, °C): |

# Health monitoring and standard hygienic procedures

For routine health monitoring, immunocompetent sentinel animals were used. These “bedding sentinels” received 100% dirty bedding (i.e. used cages including nest, food and drinking bottles), and per quarter at least one of the sentinels is tested and replaced. The mouse strain used as sentinel was changed every quarter to provide a broad spectrum of different pathogen susceptibilities. Additionally to the bedding sentinels, immunodeficient colony animals were used on an annually basis for bacteriology and PCR analysis for mouse hepatitis virus, murine norovirus, parvoviruses, and Theiler´s murine encephalomyelitis virus. Furthermore, AED (exhaust air dust) material was tested for pathogens according to FELASA recommendations including rodent-specific Helicobacter spp. by qPCR analysis. Moreover, animals suffering from clinical symptoms underwent full necropsy procedures including gross pathology as well as testing for infectious agents.

As experimental mice were housed in a conventional open housing system in the experimental room, every person with access to the room was specifically instructed to the hygienic regulations. These include a quarantine time of at least 48 h prior access without contact to rodents in other animal facilities. Before access to the animal husbandry, hand disinfection and floor mat disinfection was used. The husbandry and experimental room underlie a structured daily disinfection program. Before the experimental room was entered, hygienic clothing (surgical gowns, single use gloves, single use surgical caps, single use face masks, and single use shoe covers) was put on to protect both personnel and experimental mice.
